# T cells targeted to TdT kill leukemic lymphoblasts while sparing normal lymphocytes

**DOI:** 10.1038/s41587-021-01089-x

**Published:** 2021-12-06

**Authors:** Muhammad Ali, Eirini Giannakopoulou, Yingqian Li, Madeleine Lehander, Stina Virding Culleton, Weiwen Yang, Cathrine Knetter, Mete Can Odabasi, Ravi Chand Bollineni, Xinbo Yang, Zsofia Foldvari, Maxi-Lu Böschen, Eli Taraldsrud, Erlend Strønen, Mireille Toebes, Amy Hillen, Stefania Mazzi, Arnoud H. de Ru, George M. C. Janssen, Arne Kolstad, Geir Erland Tjønnfjord, Benedicte A. Lie, Marieke Griffioen, Sören Lehmann, Liv Toril Osnes, Jochen Buechner, K. Christopher Garcia, Ton N. Schumacher, Peter A. van Veelen, Matthias Leisegang, Sten Eirik W. Jacobsen, Petter Woll, Johanna Olweus

**Affiliations:** 1 Department of Cancer Immunology, Institute for Cancer Research, Oslo University Hospital Radiumhospitalet, Oslo, Norway; 2 K.G. Jebsen Center for Cancer Immunotherapy, Institute of Clinical Medicine, University of Oslo, Oslo, Norway; 3 Department of Medicine, Huddinge Center for Hematology and Regenerative Medicine, Karolinska Institutet, Stockholm, Sweden; 4 Charité – Universitätsmedizin Berlin, Institute of Immunology, Berlin, Germany; 5 Parker Institute for Cancer Immunotherapy, Howard Hughes Medical Institute, Departments of Molecular and Cellular Physiology and Structural Biology, Stanford University School of Medicine, Stanford, CA USA; 6 Division of Molecular Oncology & Immunology, Oncode Institute, The Netherlands Cancer Institute, Amsterdam, the Netherlands; 7 Center for Proteomics and Metabolomics, Leiden University Medical Center, Leiden, the Netherlands; 8 Department of Oncology, Oslo University Hospital Radiumhospitalet, Oslo, Norway; 9 Department of Haematology, Oslo University Hospital and KG Jebsen Center for B cell malignancies, Institute of Clinical Medicine, University of Oslo, Oslo, Norway; 10 Department of Medical Genetics, University of Oslo and Oslo University Hospital, Oslo, Norway; 11 Department of Hematology, Leiden University Medical Center, Leiden, the Netherlands; 12 Department of Medical Sciences, Uppsala University Hospital, Uppsala, Sweden; 13 Department of Immunology, Oslo University Hospital, Oslo, Norway; 14 Department of Pediatric Hematology and Oncology, Oslo University Hospital, Oslo, Norway; 15 Department of Immunohematology and Bloodtransfusion, Leiden University Medical Center, Leiden, the Netherlands; 16 David and Etta Jonas Center for Cellular Therapy, The University of Chicago, Chicago, IL USA; 17 German Cancer Consortium, partner site Berlin, Berlin, Germany; 18 German Cancer Research Center, Heidelberg, Germany; 19 Department of Cell and Molecular Biology, Karolinska Institutet, Stockholm, Sweden; 20 Karolinska University Hospital, Stockholm, Sweden; 21 MRC Molecular Haematology Unit, MRC Weatherall Institute of Molecular Medicine, University of Oxford, Oxford, UK

**Keywords:** Cancer immunotherapy, Acute lymphocytic leukaemia, Translational research, Preclinical research

## Abstract

Unlike chimeric antigen receptors, T-cell receptors (TCRs) can recognize intracellular targets presented on human leukocyte antigen (HLA) molecules. Here we demonstrate that T cells expressing TCRs specific for peptides from the intracellular lymphoid-specific enzyme terminal deoxynucleotidyl transferase (TdT), presented in the context of HLA-A*02:01, specifically eliminate primary acute lymphoblastic leukemia (ALL) cells of T- and B-cell origin in vitro and in three mouse models of disseminated B-ALL. By contrast, the treatment spares normal peripheral T- and B-cell repertoires and normal myeloid cells in vitro, and in vivo in humanized mice. TdT is an attractive cancer target as it is highly and homogeneously expressed in 80–94% of B- and T-ALLs, but only transiently expressed during normal lymphoid differentiation, limiting on-target toxicity of TdT-specific T cells. TCR-modified T cells targeting TdT may be a promising immunotherapy for B-ALL and T-ALL that preserves normal lymphocytes.

## Abstract (web summary)

Engineered T cells kill leukemic cells with little off-target toxicity.

## Main

CD19 chimeric antigen receptor (CAR) T-cell therapy frequently induces complete remission in acute B-lymphoblastic leukemia (B-ALL), dramatically improving the outcome for this patient group. However, approximately 40–50% of patients relapse^1,2^, most frequently due to loss of CD19 (refs. ^3–5^). CAR T-cell therapy directed at other B-cell-specific cell-surface molecules has shown a higher tendency to relapse^6^, possibly because of lower expression levels than CD19 on leukemia-propagating cells. Cell-based immunotherapies targeting alternative molecules expressed early during B-cell differentiation would thus be desirable. In spite of a multitude of T-cell-specific markers, no CAR therapy is approved for T-ALL that efficiently targets malignant cells across all T-ALL subtypes while at the same time sparing mature, normal T cells. This would be crucial to avoid depletion of normal T cells, which is prohibitively toxic or even incompatible with life. Moreover, no tumor-specific target has been identified in T-ALL and no immunotherapy has yet proven effective in clinical trials. In the event of failed chemotherapy (15–20%), T-ALL has a dismal prognosis with overall survival <25% (refs. ^7,8^).

**Fig. 1:**
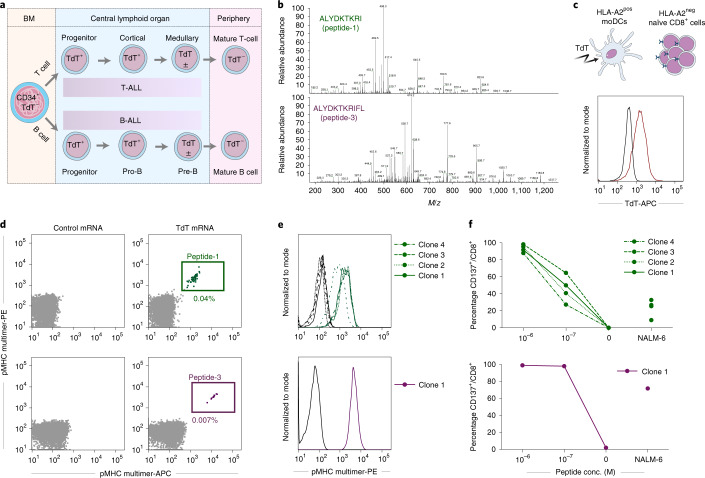
Discovery of CD8^+^ T-cell clones reactive to TdT peptides presented on HLA-A2. **a**, Illustration of TdT expression during lymphoid differentiation. **b**, Mass to charge (*m/z*) ratio spectra of TdT peptide-1 and -3. **c**, Intracellular staining of TdT in HLA-A2^pos^ moDCs after electroporation with mRNA encoding full-length TdT (red) or irrelevant control mRNA (black). HLA-A2^pos^ moDCs were then cocultured with HLA-A2^neg^ naïve CD8^+^ cells. **d**, Staining of CD8^+^ T cells with pMHC multimers complexed with peptide-1 or -3 (each multimer conjugated to both APC and PE, gating strategy shown in Extended Data Fig. 1a) following cocultures with moDCs transfected with TdT or control mRNA. **e**, Staining of T-cell clones reactive to peptide-1 (clones 1–4) and peptide-3 (clone 1) showing the relevant pMHC multimers (green and purple) and corresponding nonrelevant pMHC multimers (black). **f**, Upregulation of CD137 on T-cell clones reactive to peptide-1 (green, clones 1–4) and peptide-3 (purple, clone 1) following coincubation with TdT^neg^HLA-A2^pos^ EBV-LCL cells pulsed with indicated concentrations of cognate peptides, or the B-ALL cell line NALM-6, naturally positive for TdT and HLA-A2. conc., concentration. Source data

We hypothesized that the enzyme terminal deoxynucleotidyl transferase (TdT) might be an ideal target of immunotherapy in B-ALL and T-ALL, for several reasons. First, as the function of TdT is to add N-nucleotides to the V-D-J junctions during recombination of the TCR and B-cell receptor^9^, its expression is confined to the B and T lineages. Second, TdT is overexpressed in 80–94% of ALL and lymphoblastic lymphoma of B- and T-cell origin^10–12^, making it a widely used diagnostic marker^13^. Third, expression of TdT is restricted to a narrow window during differentiation of early B- and T-cell progenitors in bone marrow (BM) and thymus, and it is not expressed in hematopoietic stem cells^14^. Therefore, it is expected that both naïve and mature B and T lymphocytes, as well as myeloid cells, would be spared^15^ (Fig. 1a). However, since TdT is localized intracellularly it cannot be targeted by a CAR. In contrast to CARs, TCRs can recognize intracellular antigens presented on the cell surface in complex with major histocompatibility complex (MHC) molecules. T cells that bind self-antigens with high affinity are, however, depleted from the autologous repertoire during thymic negative selection. To circumvent this, we used a technology whereby our previously published protocol for identification of healthy donor T cells that recognize foreign neoantigens presented on self-HLA^16^ was modified to allow for selection of high-affinity T cells reactive to self-antigen presented in the context of foreign HLA. We identified TCRs recognizing TdT-derived peptides presented on HLA-A*02:01 (HLA-A2), expressed in approximately 50% of people of European, Middle Eastern or North African ancestry. We also evaluated the safety and efficacy of these TCRs following treatment of leukemia with TCR-engineered T cells in vitro and in vivo.

## Results

**Fig. 2:**
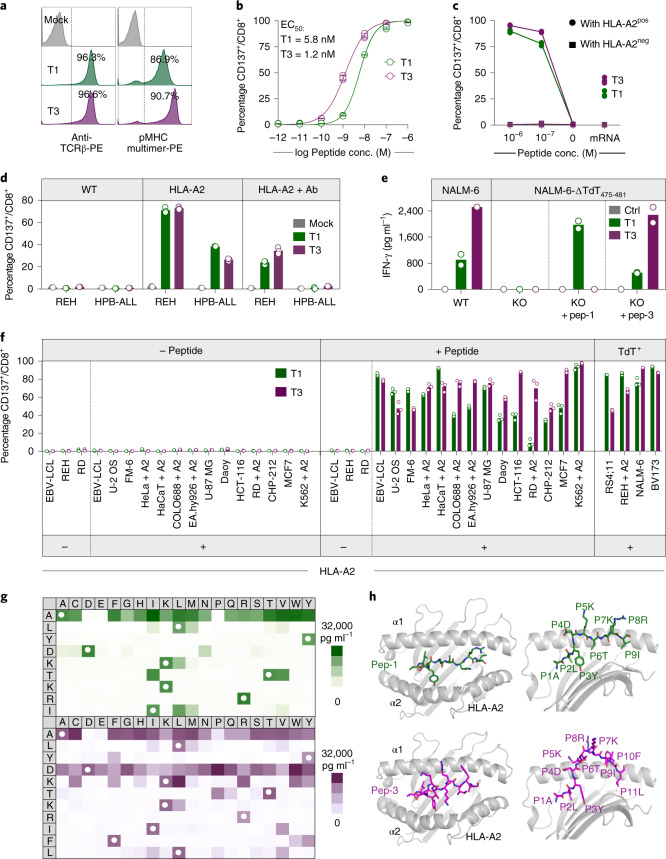
T1 and T3 are specific for TdT and HLA-A2 and do not show off-target reactivity. **a**, Histograms of viable CD8^+^ T cells transduced with T1 and T3 TCRs stained with anti-mouse TCR-β antibody or relevant peptide–MHC multimers. **b**, Activation of T1 and T3 cells following coincubation with peptide-pulsed T2 lymphoblast cells. Data are pooled from three independent experiments, with each circle representing the mean of three technical replicates in each experiment. Data are shown as mean ± s.d. **c**, CD137 upregulation on CD8^+^ T1 (green) and T3 (purple) cells after coculture with EBV-LCLs derived from one HLA-A2^pos^ and one HLA-A2^neg^ donor, either pulsed with relevant peptides or electroporated with mRNA encoding full-length TdT. **d**, Activation of CD8^+^ T1 and T3 cells after coculture with TdT^pos^ cell lines REH (B-ALL) and HPB-ALL (T-ALL) in the presence/absence of introduced expression of HLA-A2, or pan-MHC class I blocking antibody W6/32 (Ab). **e**, IFN-γ production by T1 and T3 cells following coincubation with TdT^pos^ NALM-6 cells (WT), or NALM-6 cells in which TdT_475-481_ was deleted by CRISPR–Cas9 (KO) in the presence/absence of added peptide. **f**, Activation of T1 and T3 cells after coculture with various cell lines, with indicated HLA-A2 and TdT expression, loaded or not with TdT peptides (2 × 10^−7^ M). The suffix + A2 denotes cell lines transduced with HLA-A*02:01. **c**–**f**, Results are from one experiment representative of two or three performed with different T-cell donors. Bars or connecting lines show mean, and individual data points represent either two (**e**) or three (**c**,**d**,**f**) technical replicates. **g**, Heat maps of IFN-γ produced by T1 (green) and T3 cells (purple) coincubated with EBV-LCLs pulsed with peptides from mimotope libraries. White circles, amino acid in wild-type peptide. IFN-γ concentration range for positive reactions was 500–31,254 pg ml^–1^. One replicate per condition (see Extended Data Fig. 2c for correlation with CD137 activation assay). **h**, Model structures of TdT peptide-1 (green) and -3 (purple) represented as sticks, bound to the HLA-A2 molecule, in gray, shown from top (left) and side views (right). Individual amino acids are labeled with positional number and symbol. Source data

**Extended Data Fig. 1:**
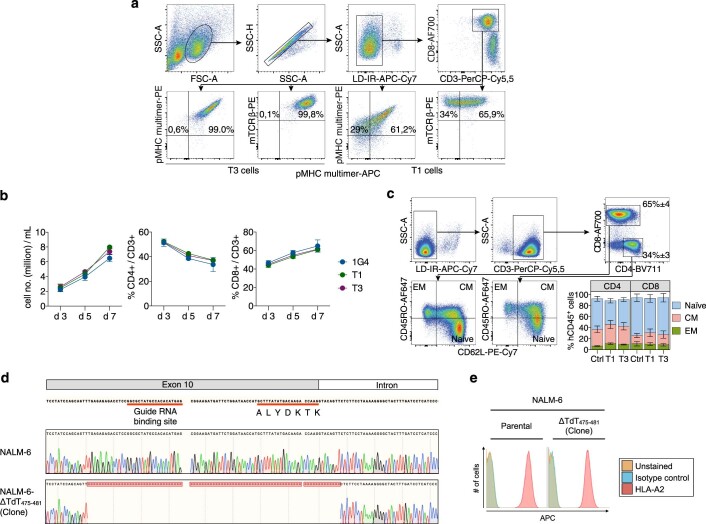
T1 and T3 cells maintain a predominantly naïve/CM phenotype following expansion. (a) Gating strategy to analyze TCR transduced T cells (percentage of parental gate is shown). Top four panels: gating on FSC/SSC^high^, Live/Dead Fixable Near-IR^neg^, CD8^+^ singlets. Lower four panels: cells staining positively for APC and PE-labeled pMHC multimers complexed with either peptide-3 (left two plots: T3-transduced cells) or peptide-1 (right two plots: T1-transduced cells) and anti-mouse TCR-β antibody. Although >90% of T1 cells stain positively for the pMHC-PE multimer, a lower % was positive for the corresponding APC multimer due to lower fluorescence intensity of APC relative to PE. (b) Expansion of PB T cells after transduction with T1, T3 or 1G4 at indicated days after retroviral transduction. Data shown as mean ± s.e.m. of *n* = 3 (d3 and d7) or *n* = 2 (d5) HLA-A2^pos^ donors. (c) FACS plots showing phenotyping of T cells transduced with T1 on day 7 after spinoculation, gating on FSC/SSC^high^, Live/Dead Fixable Near-IR^neg^, CD3^+^ singlets, that are either CD4^+^ or CD8^+^. Naïve, CM (central memory) and EM (effector memory) T cells. Bar graph in the lower right corner shows proportion of naïve, CM and EM CD4^+^ or CD8^+^ T cells on d7 after retroviral transduction with T1, T3 or control TCR. Inset numbers in the top right plot represent mean ± s.e.m. of all groups analyzed together due to similar proportions of CD8^+^ and CD4^+^ T cells in experimental groups. Data shown as mean ± s.e.m. of *n* = 3 donors. (d) Top: The region of gDNA in exon 10 which ends with the first 7 amino acids of the A2-presented epitope, selected as binding site for guide RNA. Middle: sequencing of gDNA from wild type NALM-6 cells, in which the TdT sequence corresponds to the sequence deposited in the database. Bottom: after cloning of CRISPR/Cas9-modified NALM-6 cultures, the selected clone shows a clean deletion in both alleles of the TdT gene, including the first 7 amino acids of the A2-presented epitope. (e) HLA-A2 expression on NALM-6 cells with TdT knock out (right) is unaffected relative to parental cell line (left). Source data

We determined the sequence of TdT-derived peptides that were naturally processed and presented on HLA-A2 from HLA-A2^pos^ Epstein–Barr virus-transformed lymphoblastoid cells (EBV-LCL) transduced with full-length TdT, by mass spectrometry (MS). Two peptides identified as HLA-A2 binders (ALYDKTKRI (peptide-1) and ALYDKTKRIFL (peptide−3)) were further characterized (Fig. 1b, Supplementary Fig. 1a,b and Supplementary Table 1). Naïve T cells from HLA-A2^neg^ donors were cocultured with monocyte-derived dendritic cells (MoDCs) generated from HLA-A2^pos^ donors that were electroporated with TdT-encoding messenger RNA (Fig. 1c)^16^. T cells staining positively with peptide–major histocompatibility complex (pMHC)-multimers (Fig. 1d) were sorted as single cells to generate T-cell clones. Clones reactive to peptide-1 and peptide-3 stained positively with the relevant multimers (Fig. 1e) and were activated by peptide-pulsed HLA-A2^pos^ EBV-LCL, and by a B-ALL cell line endogenously expressing TdT (Fig. 1f). One TCR sequence was identified from clones reactive to each peptide, named T1 (reactive to peptide 1) and T3 (reactive to peptide-3). Both TCRs were efficiently expressed in third-party peripheral blood (PB) CD8^+^ T cells, as demonstrated by staining with either pMHC–multimers or anti-mouse TCR-β antibodies reactive to the mouse constant region introduced into the TCRs^17^ (Fig. 2a). All T3-transduced cells and the majority of T1-transduced cells that stained positively with anti-mouse TCR-β were also pMHC–multimer positive, indicating preferential pairing of introduced TCR-α and -β chains after retroviral transduction (Extended Data Fig. 1a)^18^. T cells from HLA-A2^pos^ donors that were transduced with T1 and T3 (T1 and T3 cells) expanded equally well as those transduced with the control TCR 1G4 (Extended Data Fig. 1b), specific for NY-ESO-1 and safely used in clinical trials^19^. The results indicate the absence of fratricide, as expected from lack of TdT expression in PB T cells^15^. The majority of transduced cells had a naïve or central memory phenotype (Extended Data Fig. 1c). T1 and T3 cells recognized their cognate peptides with high sensitivity (T1 half-maximal effective concentration (EC_50_) = 5.8 nM and T3 EC_50_ = 1.2 nM; Fig. 2b).

T1 and T3 cells recognized target cells in an HLA-A2-restricted and TdT-dependent manner. Thus, T1 and T3 cells were activated only by EBV-LCLs presenting TdT peptides introduced by either mRNA electroporation or external peptide loading when HLA-A2 was simultaneously expressed (Fig. 2c). Similarly, naturally TdT^pos^ but HLA-A2^neg^ cell lines REH (B-ALL origin) and HPB-ALL (T-ALL origin) activated T1 and T3 cells only when HLA-A*02:01 was introduced. Activation was reduced by the addition of the MHC class I blocking antibody W6/32 (Fig. 2d). Moreover, CRISPR–Cas9-mediated knockout of TdT abolished recognition of the B-ALL cell line NALM-6 (naturally TdT^pos^ and HLA-A2^pos^) by T1 and T3 cells (Fig. 2e and Extended Data Fig. 1d,e). T-cell activation was not observed when T1 and T3 cells were cocultured with a large panel of human TdT^neg^ cell lines of different tissue origin, unless loaded with relevant peptide and expressing HLA-A2, naturally or by genetic introduction (Fig. 2f). These results showed that T1 and T3 TCRs did not react with unintended targets presented on HLA-A2, or on a large variety of other HLA molecules expressed by the cell lines.

**Extended Data Fig. 2:**
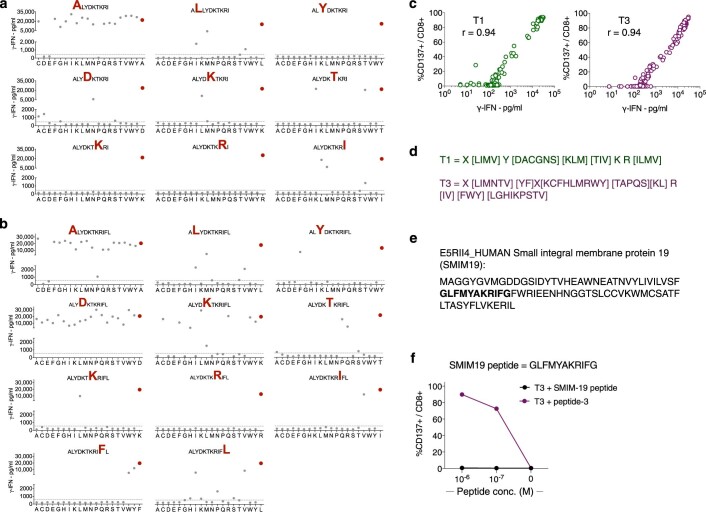
Comprehensive mapping of peptide reactivity does not reveal naturally existing peptides to which T1 or T3 cells cross-react. (a,b) Graphs depicting IFN-γ response of T1 (a) and T3 (b) cells to EBV-LCLs loaded with individual peptides from mimotope libraries containing a total of 161 9-mers for peptide-1 and 201 11-mers for peptide-3, at a concentration of (2 x 10^−7^ M). Red dot in each graph represents response to wild-type peptide. Substituted amino acid in the original peptide is highlighted in red in the graph heading. IFN-γ concentration range for positive reactions was 500 – 31254 pg/mL (cut-off indicated by horizontal lines, one replicate per condition). (c) Correlation analysis between data for IFN-γ release, and percentage of cells expressing CD137, by T1 (left) and T3 cells (right) following activation by EBV-LCLs loaded with individual peptides from the mimotope libraries. r = Pearson correlation coefficient. (d) Peptide reactivity motifs for T1 and T3 that were queried in the ScanProsite search tool against human proteome databases. X indicates that any amino acid is allowed, while amino acids in square brackets [] indicate alternatives that are allowed for that given position in the peptide motifs. (e) Amino acid sequence of the human small integral membrane protein 19 (SMIM19), as listed in the non-curated TrEMBL database. (f) Percentage CD137^+^ events among T3 CD8^+^ T cells after 18 h co-culture with target cells loaded with indicated concentrations of either peptide-3 or SMIM19 peptide. Data are shown as mean of three technical replicates from one experiment representative of 2 performed. Source data

**Extended Data Fig. 3:**
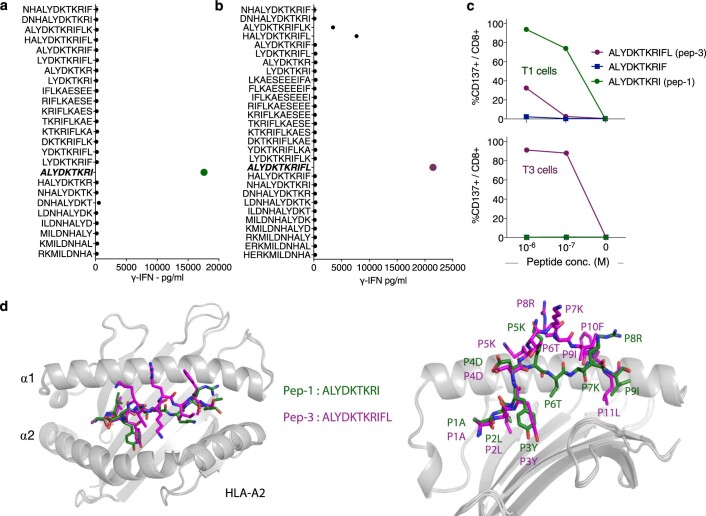
T1 and T3 cell activation in response to length variants of cognate peptides and model of structural conformation of TdT peptides in the HLA-A2 peptide-binding groove. (a,b) IFN-γ production by the T1 (a) and T3 (b) cells after co-culture with target cells loaded with indicated peptides. 9-mer and 11-mer peptides were included that contained amino acids upstream or downstream of the wild type peptide-1 and -3 in the TdT protein sequence. In addition, 8 to 12 amino acid long peptides were included that contained parts or all of peptide-1 and -3. One replicate per condition. (c) Percentage of CD137^+^ events among T1 and T3 cells after co-culture with EBV-LCLs loaded with indicated concentrations of cognate, or non-cognate, peptide-1 (ALYDKTKRI) or -3 (ALYDKTKRIFL). Reactivity to a 10-mer peptide (ALYDKTKRIF) is also shown. One replicate per condition. Data shown are from one experiment representative of 2 performed. (d) Overlaid structural conformations of peptide-1 and -3 bound to the HLA-A2 molecule shown from top (left) and side view (right). HLA-A2 is shown as cartoon in gray. Peptide-1 (green) and peptide-3 (purple) are shown as stick. Individual amino acids are labeled as a number in the corresponding peptide followed by their symbol. The modeled peptide-1 on HLA-A2 displays a flattened conformation, and amino acids at position (P)4, P5, P7 and P8 are facing up for potential TCR engagement. The model is supported by the mimotope reactivity pattern, as T1 was intolerant to mutations in P4, P5, P7 and P8. Although P3Y is not facing upward for TCR contact, it is likely to stabilize the peptide-MHC interaction and is thus sensitive to mutations. The P4, P5, P7 and P8 as core TCR contact residues are salient features for 9-mer peptides presented by HLA-A2. Peptide-3 displays a bulged conformation, in which P4, P5, P6, P7, P8, P9 residues are facing upwards for TCR contact. P4D and P5K are less important for TCR engagement than for peptide-1, as P4D can be substituted by almost any amino acid and P5K can be substituted by amino acids with long or bulky side chains. P6-P10 are potentially important for TCR contact, and as shown in our cross-reactivity assay, T3 was less tolerant for substitutions of these amino acids. Source data

Precise mapping of TCR reactivity can inform about cross-reactivity of potential clinical importance. To this end, we synthesized peptide mimotope libraries in which each amino acid residue of peptide-1 or -3 was replaced by all other natural amino acids, one at a time. T1 and T3 cells were combined with target cells loaded with single mimotopes from the relevant library. Resultant T-cell activation, as measured by production of IFN-γ or upregulation of CD137, was highly correlated (Fig. 2g and Extended Data Fig. 2a–c). Next, we queried the curated the human proteome databases UniProtKB/Swiss-Prot and Protein Data Bank for all combinations of amino acid substitutions that induced reactivity in T1 or T3 cells, by employing the ScanProsite tool (https://prosite.expasy.org/scanprosite/) (Extended Data Fig. 2d). The search did not identify any naturally occurring 9- or 11-mer peptide in the human proteome that matched these combinations. When searching a noncurated database (UniProtKB/TrEMBL), one peptide derived from the ubiquitously expressed small integral membrane protein 19 matched reactivity combinations for T3 but did not induce any response in T3 cells (Extended Data Fig. 2e,f). Taken together, the data provided no evidence for off-target reactivities by T1 and T3. Moreover, peptides prolonged upstream and/or downstream of peptide-1 and -3 sequences in the TdT protein failed to activate T1 and T3 cells, unless longer variants encompassing the whole cognate peptide sequence were used (Extended Data Fig. 3a,b). Furthermore, peptide-1 failed to activate T3 cells whereas peptide-3 activated T1 cells only at high concentrations, probably due to the breakdown of peptide-3 generating peptide-1 in culture (Extended Data Fig. 3c). Lack of cross-activation might be explained by a large difference in the structural configuration of the two peptide–MHC complexes modeled in Fig. 2h and Extended Data Fig. 3d. Peptide-1 adopts a flattened conformation in the HLA-A2 peptide groove whereas peptide-3 displays a bulging conformation. To accommodate this, T3 is likely to be ‘lifted up’ and make more interactions with peptide-3 but less contact with MHC, while T1 makes more evenly distributed interactions with peptide-1 and MHC.

**Fig. 3:**
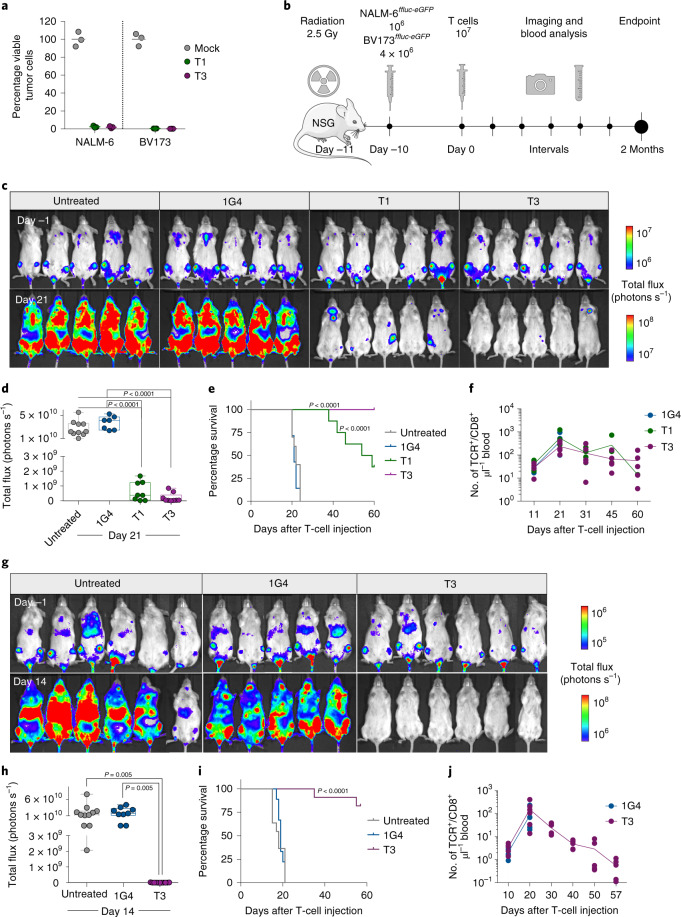
T1 and T3 cells efficiently kill leukemia cells in vivo. **a**, Viable TdT^pos^HLA-A2^pos^ NALM-6 and BV173 cells after 48 h of coculture with T1 and T3 cells (E/T ratio, 1/1), in percentage of corresponding numbers following treatment with mock-transduced T cells as quantified by flow cytometry. Data points represent technical replicates in one experiment representative of three performed. **b**, Experimental overview of BV173 and NALM-6 in vivo models. BLI (**c**) and quantification (**d**) of BV173-bearing mice 1 day before and 21 days after treatment with human T cells transduced with 1G4, T1 or T3, or left untreated. Survival analysis (**e**) and numbers (**f**) of TCR-transduced CD8^+^ T cells μl^–^^1^ blood at indicated time points in the aforementioned experimental groups. BLI (**g**) and quantification (**h**) of NALM-6-bearing mice 1 day before and 14 days after treatment with human T cells transduced with 1G4, T3, or left untreated. Survival analysis (**i**) and numbers (**j**) of TCR-transduced CD8^+^ T cells μl^–^^1^ blood at indicated time points in the aforementioned experimental groups. **d**,**e**,**h**,**i**, Data are pooled from two independent experiments: untreated, *n* = 10 (BV173) or *n* = 11 (NALM-6); 1G4, *n* = 7 (BV173) or *n* = 9 (NALM-6); T1, *n* = 8 (BV173); T3, *n* = 9 (BV173) or *n* = 11 (NALM-6). **d**,**h**, Box plots showing interquartile range (25th–75th percentile) with central bar indicating the median and whiskers indicating the range. Dots represent individual mice and data analyzed by one-way ANOVA with adjustment for multiple comparisons with Tukey’s post-test. **e**,**i**, Survival analysis performed by two-sided log-rank (Mantel–Cox) test. **f**,**j**, Data shown are from one representative experiment out of two performed. *n* = 5–6 mice per group. Connecting lines, mean; dots, individual mice. Source data

**Extended Data Fig. 4:**
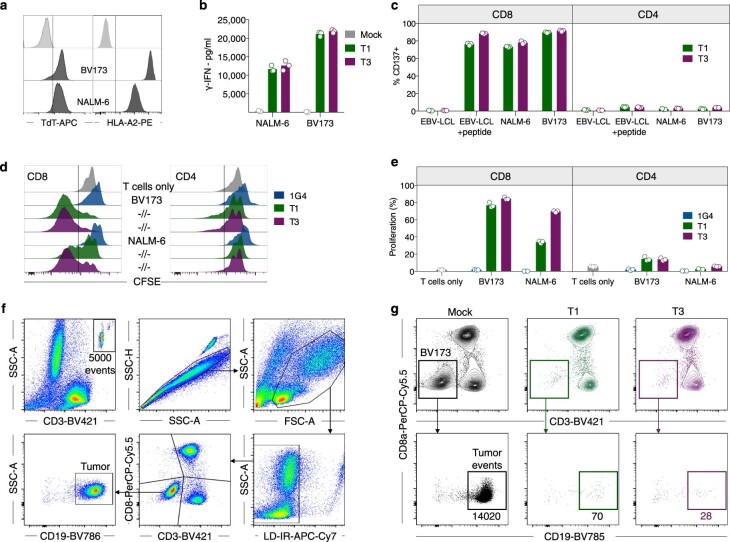
T1 and T3 cells are activated by, and effectively kill, TdTpos HLA-A2pos leukemia cell lines. (a) Flow cytometry histograms showing natural TdT or HLA-A2 expression in the B-ALL cell lines NALM-6 and BV173. (b) IFN-γ production in the co-culture supernatants of T1, T3 or mock T cells with indicated cell lines as measured by ELISA. (c) Expression of CD137 measured by flow cytometry by T1 and T3 cells after 18 h co-culture with indicated cell lines, loaded or not with relevant peptide and gated on CD8^+^ or CD4^+^ T cells. (d,e) Proliferation of PB T cells following transduction of T1, T3 or 1G4 in response to NALM-6 and BV173 following 5 days of co-culture at an E/T ratio of 1/1. Percentage proliferating cells is calculated based on events with low Cell Trace Violet staining out of total events that are FSC/SSC^high^, Live/Dead Fixable Near-IR^neg^, CD3^+^ singlets staining positively for either CD8^+^ or CD4^+^. Bar graphs in (b), (c) and (e) show mean of three technical replicates from one experiment representative of 2 or 3 performed. (f) Gating strategy for the flow cytometry-based killing assay to enumerate BV173 tumor cells, here co-cultured with mock-transduced cells. Fluorescent beads (10,000) were added into each well and 5,000 beads were acquired for flow cytometry analysis. Live BV173 tumor cells were identified as Live/Dead Fixable Near-IR^neg^ singlet cells that were CD3^−^, CD8^−^ and CD19^+^. (g) Flow cytometry plots of BV173 cells co-cultured with mock (black), T1 (green) and T3 cells (purple) for 48 h. Inset numbers display event counts within the live tumor cell gate. Source data

**Extended Data Fig. 5:**
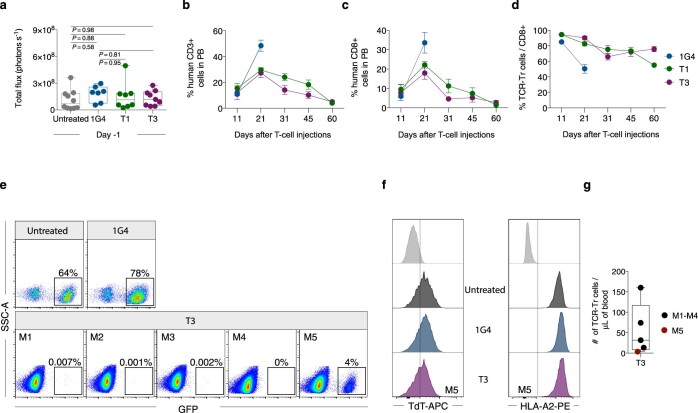
T1 and T3 cells proliferate and control BV173 ALL in vivo. (a) Bioluminescence imaging (BLI) analysis of NSG mice on day 9 after BV173^*ffluc-eGFP*^ cell injection, one day prior to T-cell therapy. Data were pooled from two independent experiments, untreated *n* = 10, 1G4 *n* = 7, T1 *n* = 8, T3 *n* = 9. Data analysis by one-way ANOVA with Tukey’s multiple comparison test. (b,c) The percentage of human CD3^+^ (b) and CD8^+^ cells (c) in PB of mice analyzed at indicated time points out of total CD45^+^ human and CD45^+^ mice leukocytes. (d) Percent cells expressing T1, T3 and 1G4 TCRs among human CD8^+^ T cells throughout the experiment. Data in b-d are shown as mean ± s.e.m., *n* = 5/group. (e) Flow cytometry plots showing bone-marrow tumor burden in five T3-treated mice analysed on day 60 (M1-M5), compared to the 1G4-treated or untreated mouse at time of sacrifice (day 21). Threshold for positive leukemia detection was set as GFP^+^ cells ≥ 0.01% of live viable cells. (f) Expression of HLA-A2 and TdT in leukemia cells harvested from the bone marrow of untreated, 1G4 and the one T3-treated mouse with detectable tumor burden (M5). Light gray histograms represent negative control. (g) Numbers of TCR-transduced CD8^+^ cells in blood of T3-treated mice on day 60 (*n* = 5, M1-4: black circles, M5: red circle). Box plots in (a) and (g) show interquartile range (25^th^ to 75^th^ percentile) with central bar indicating the median and whiskers indicating the range while dots represent data from individual mice. Source data

**Extended Data Fig. 6:**
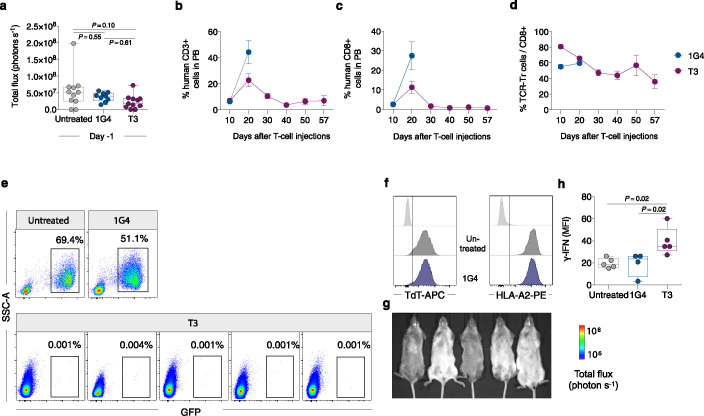
T3 cells proliferate and control NALM6 ALL in vivo. (a) BLI analysis of NSG mice on day 9 after NALM-6^*ffluc-eGFP*^ cell injection, one day prior to T-cell therapy. Data were pooled from two independent experiments, untreated *n* = 11, 1G4 *n* = 9, T3 *n* = 11. Data analysis by one-way ANOVA with Tukey’s multiple comparison test. (b,c) The percentage of human CD3^+^ (b) and CD8^+^ cells (c) in peripheral blood (PB) of mice analyzed at indicated time points out of total CD45^+^ human and CD45^+^ mouse leukocytes. (d) Percent cells expressing T3 and 1G4 TCRs among human CD8^+^ T cells throughout the experiment. Data in b-d are shown as mean ± s.e.m., *n* = 5 (1G4) and *n* = 6 (T3). (e) Flow cytometry plots showing bone-marrow tumor burden in one untreated and one 1G4-treated mice at time of sacrifice (day 14), and in five T3-treated mice sacrificed at end of experiment (day 57). (f) Expression of HLA-A2 and TdT in leukemia cells detected in bone marrow of untreated and 1G4-treated mice at the time of sacrifice. Light gray histograms represent negative control. (g) BLI images of mice in T3-treated group on day 57. Two of the T3-treated mice died for unknown reasons on day 35 and day 55 after T cell therapy, following negative BLI and PB flow cytometry analysis 5 days earlier, respectively, indicating that death was not related to leukemia. (h) IFN-γ levels in sera from untreated (*n* = 5), 1G4 (*n* = 4) or T3 (*n* = 5) cell-treated mice on day 2 after T cell therapy, measured as mean fluorescence intensity (MFI) of IFN-γ capturing fluorescent beads. Statistical analysis by one way ANOVA with Tukey’s multiple comparison test. Box plots in (a) and (h) show interquartile range (25^th^ to 75^th^ percentile) with central bar indicating the median and whiskers indicating the range, while dots represent data from individual mice. Source data

T1 and T3 cells responded strongly to B-cell leukemia cell lines BV173 and NALM-6 (naturally TdT^pos^ and HLA-A2^pos^), as measured by the production of IFN-γ, proliferation and killing (96–99% at an effector/target (E/T) ratio of 1/1) (Fig. 3a and Extended Data Fig. 4a–g). To study the efficacy in vivo, we engrafted BV173^*ffluc-eGFP*^ cells or NALM-6^*ffluc-eGFP*^ cells in NOD-*scid* IL2Rg^null^ (NSG) mice and started treatment with T1 or T3 cells after leukemia establishment (Fig. 3b and Extended Data Figs. 5a–d and 6a,d). A very low (T1) or no (T3) tumor signal was observed in these mice on days 21 (BV173; Fig. 3c,d) and 14 (NALM-6, Fig. 3g,h), shortly after which untreated and 1G4-treated control mice had to be sacrificed because of high tumor burden (Fig. 3e,i). Notably, none of the mice treated with T3 cells died from leukemia during the observation period following injection of leukemic cells (Fig. 3e,i). Two T3 cell-treated mice in the NALM-6 model died from reasons unknown and not related to leukemia spread. Bioluminescence imaging (BLI) remained negative on day 57 in the T3 cell-treated mice in the NALM-6 model, consistent with absence of tumor in the BM of sacrificed mice (Extended Data Fig. 6e–g). Only one out of five T3-treated BV173 mice had green fluorescent protein (GFP)^+^ tumor cells in the BM upon sacrifice, probably due to reduced T-cell numbers and not related to antigen downregulation (Extended Data Fig. 5e–g). Mice treated with T3 cells had higher IFN-γ levels in serum on day 2 after adoptive T-cell transfer as compared with those treated with 1G4 T cells (Extended Data Fig. 6h). TCR-transduced T cells were still detected in peripheral blood at the experimental endpoint (Fig. 3f,j and Extended Data Figs. 5b–d and 6b–d), although their numbers decreased after tumor eradication. Control 1G4 TCR-transduced T cells also expanded initially, probably due to injections of IL-2. Survival benefit was highly significant also for BV173 mice treated with T1 cells (Fig. 3e), but several of these mice eventually succumbed to tumor, consistent with the lower peptide sensitivity for T1 as compared with T3.

**Fig. 4:**
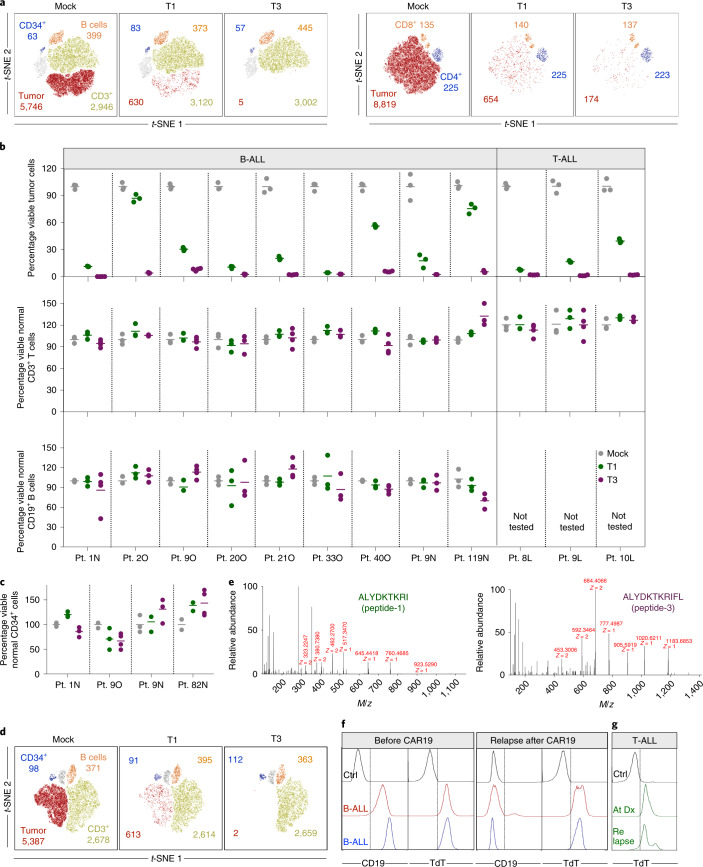
T1 and T3 cells deplete patient-derived cancer cells while sparing normal B and T lymphocytes and nonlineage-committed hematopoietic progenitor cells. **a**, Representative *t*-SNE plots showing live HLA-A2^pos^, TdT^pos^ B-ALL tumor cells (CD19^+^CD10^+^ events, left) and T-ALL tumor cells (CD5^+^CD7^+^CD99^+^ and surface CD3^−^CD4^−^ events, right), normal B cells (CD19^+^CD10^−^), normal T cells (CD3^+^ and CD8^+^ or CD4^+^) and CD34^+^lin^−^ progenitor cells following 72 h of coculture with mock-, T1- or T3-transduced T cells (E/T ratio, 1/1), as quantified by flow cytometry. **b**, Diagnostic samples from 12 patients (Pt.) with HLA-A2^pos^, TdT^pos^ B-ALL or T-ALL, assayed as described in **a**. Each dot represents the number of live tumor, normal B or T cells after coculture with T1 (green) or T3 (purple) cells, as percentage of corresponding numbers in cultures treated with mock-transduced T cells (gray). **c**, Dots showing numbers of live CD34^+^lin^−^ cells after coculture with T1 (green) and T3 (purple) cells as percentage of corresponding numbers in cocultures treated with mock-transduced T cells (gray). **b**,**c**, Data points represent three or four technical replicates and horizontal lines denote mean. Data shown are from one experiment representative of at least two performed for each patient sample. **d**, *t*-SNE plot of PB diagnostic sample from B-ALL patient no. 1N after 72 h of coculture with autologous T cells transduced with T1, T3 or mock. **a**,**d**, Inset numbers denote absolute event counts of the indicated cell populations. **e**, Tandem mass spectrometry fragmentation spectra of TdT peptide-1 and -3 identified from leukemia cells of patient no. 119N. **f**, Expression of CD19 and TdT in leukemia cells before and after relapse from CD19-specific CAR T-cell therapy in two patients with B-ALL. Ctrl, control. **g**, Expression of TdT in leukemia cells from a patient with T-ALL at the time of primary diagnosis (Dx) and at relapse after chemotherapy. Source data

**Extended Data Fig. 7:**
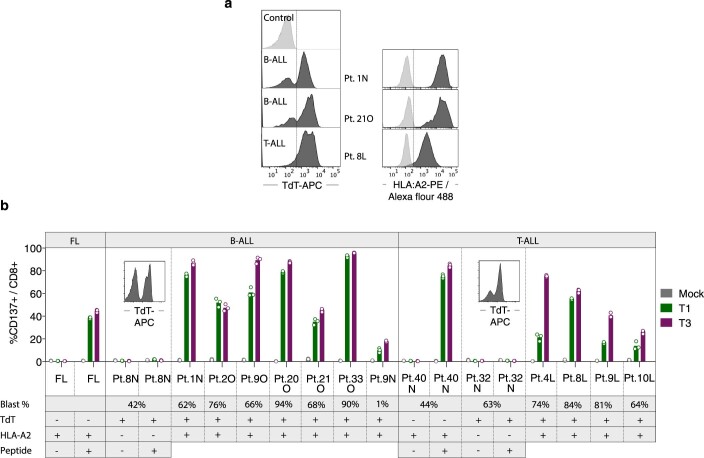
Primary patient B-ALL and T-ALL cells express TdT and activate T1 and T3 cells. (a) TdT and HLA-A2 expression in diagnostic samples (PBMC) from three representative patients with B-ALL and T-ALL as analyzed by flow cytometry. Cytotoxicity data for these patients are shown in Fig. 4a, b. (b) Percentage of CD137^+^ events among T1, T3 and mock-transduced CD8^+^ cells after 18 h culture with primary B-ALL and T-ALL patient samples that were either HLA-A2^pos^ TdT^neg^, HLA-A2^neg^ TdT^pos^ or HLA-A2^pos^ TdT^pos^. Single positive patient samples were loaded or not with peptide-1 or -3 (concentration 10^−7^ M) for 1 h before co-culture with corresponding T1 or T3 cells. FL: Follicular lymphoma, included as an HLA-A2^pos^ TdT^neg^ control. Numbers below patient index show percentages of malignant blasts in the patient sample as analyzed by flow cytometry. Bar graphs in (b) show mean of 2-3 technical replicates run for individual patients. Source data

**Extended Data Fig. 8:**
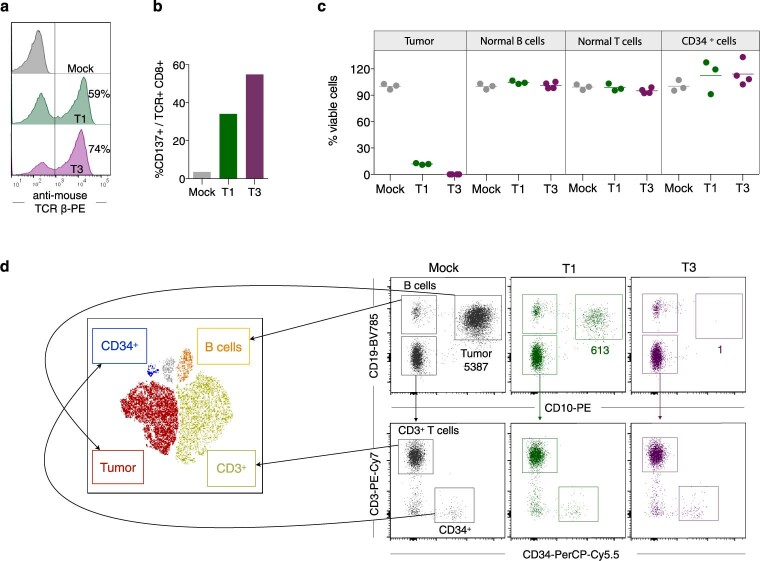
Patient-derived CD8^+^ T cells transduced with T1 or T3 efficiently kill autologous B-ALL cells. (a) Transduction efficiency of the T1 (green) and T3 (purple) TCRs in normal CD8^+^ T cells from an HLA-A2^pos^ TdT^pos^ patient diagnosed with B-ALL. Histograms are gated on live, CD8^+^ T cells. (b) T1 and T3 cell activation after 18 h co-culture with the autologous blasts. (c) Quantification of malignant cells, normal B, T and CD34^+^lin^−^ progenitor cells after performing the flow cytometry-based cytotoxicity assay for 72 h. Data points represents technical replicates (3-4) from one representative experiment out of 2 performed and horizontal lines show mean. (d) Flow cytometry dot plots from one of the test replicates in c, showing gating strategy to identify tumor blasts (CD19^+^CD10^+^), normal B cells (CD19^+^CD10^−^), T cells (CD19^−^CD10^−^CD3^+^) and CD34^+^ progenitor cells (CD19^−^CD10^−^CD3^−^CD34^+^). Populations were gated and overlaid on a t-SNE plot with designated colors as indicated (left view). Inset numbers in d show event count of tumor cells after co-culture with mock, T1 and T3 cells. Source data

We next quantified the ability of T1 and T3 cells to selectively recognize human primary ALL cells in samples also containing normal B and T cells and nonlineage-committed hematopoietic progenitor cells. Cryopreserved TdT^pos^ and HLA-A2^pos^ diagnostic samples from nine patients with B-ALL and three with T-ALL (Extended Data Fig. 7a and Supplementary Table 2) were cocultured with T1 or T3 cells. Following 48–72 h of coculture, T3 cells eliminated on average 97%, and T1 cells 69%, of leukemic blasts (T3, mean 97%, range 92–99.9%; T1, mean 69%, range 13–96%, *n* = 12). In contrast, normal B and T cells remained unaffected (Fig. 4a,b) as did noncancerous CD34^+^lin^−^ cells detected in four patients (Fig. 4a,c). Moreover, T1 and T3 cells were activated only by HLA-A2 and TdT double-positive patient leukemia cells. HLA-A2^neg^ TdT^pos^ malignancies failed to activate T1 and T3 cells, even in the presence of exogenously loaded peptide, while TdT^neg^ HLA-A2^pos^ primary follicular lymphoma and T-ALL cells activated T1 and T3 cells only when loaded with TdT peptides (Extended Data Fig. 7b). We next showed that the introduction of T3 into normal T cells from a patient with HLA-A2^pos^ TdT^pos^ B-ALL resulted in T-cell activation and elimination of virtually all autologous tumor cells. Normal B, T and CD34^+^lin^−^ progenitor cells were spared (Fig. 4d and Extended Data Fig. 8). To demonstrate directly that the results above reflect the presentation of peptides-1 and -3 on patient-derived leukemia cells, we eluted peptides from HLA-A2 molecules of patient 119N, as well as from BV173 cells, and identified the sequences by MS (Fig. 4e and Supplementary Fig. 2). Taken together, these data indicate a high degree of target selectivity and therapeutic efficacy of T1 and T3 cells against patient-derived B- and T-ALL cells with representative TdT and HLA-A2 expression.

Pediatric patients with B-ALL that were treated with CD19 CAR T-cell therapy, and subsequently relapsed with either CD19^pos^ or CD19^neg^ disease, had high levels of TdT expression in leukemic blasts both before and after CAR T-cell therapy (Fig. 4f and Supplementary Table 3). Before CAR T-cell infusion, all patients were extensively pretreated with several lines of therapy, including allogeneic stem-cell transplantation in two patients. Likewise, we observed high TdT levels in leukemic cells from a patient with T-ALL at diagnosis, at time points with measurable minimal residual disease (MRD) after chemotherapy and following relapse (Fig. 4g and Supplementary Table 4). This further suggested that TdT-targeting TCRs might represent an attractive therapeutic option for these patient groups with refractory and relapsed disease and a dismal prognosis.

**Fig. 5:**
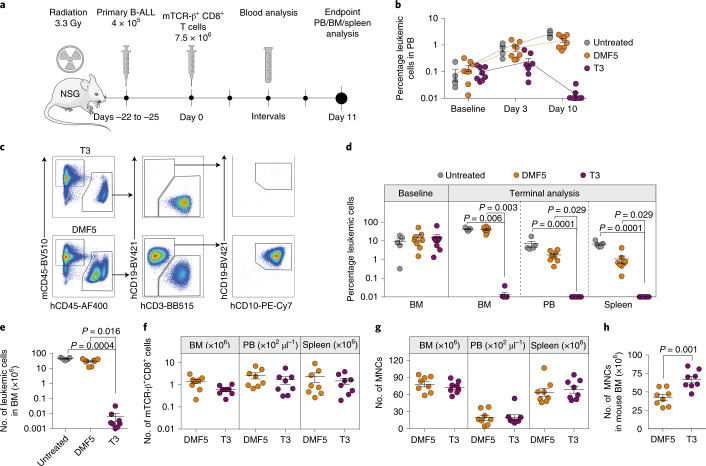
T3 cells efficiently eliminate primary B-ALL cells in vivo. **a**, Experimental overview of the PDX model. **b**, Percentage leukemic cells adjusted for human T cells in PB at baseline and at indicated time points after T-cell infusion. **c**, Representative FACS plots of viable single MNCs from BM of T3-treated (top) and DMF5-treated (bottom) NSG mice engrafted with primary human B-ALL cells. **d**, Percentage leukemic cells (hCD45^+^CD19^+^CD10^+^) adjusted for human T cells at baseline (BM) and at terminal analysis on day 11 after T-cell infusion (BM, PB and spleen). **e**, Number of leukemic cells present in BM. **f**, Number of TCR-transduced human CD8^+^ cells in BM, PB and spleen. **g**, Total numbers of human- and mouse-derived MNCs in BM, PB and spleen. **h**, Number of mouse CD45^+^ cells in BM. **b**,**d**–**h**, Data are pooled from two independent experiments and presented as mean ± s.e.m. of untreated (*n* = 5), DMF5-treated (*n* = 8) and T3-treated (*n* = 8) mice. Populations identified by flow cytometry according to gating strategy shown in **c**. Kruskal–Wallis one-way ANOVA by Dunn’s multiple comparisons test (**d**,**e**) and two-tailed Mann–Whitney test (**f**–**h**) were performed for statistical analyses. Source data

We next investigated the efficacy of T3-cell treatment on NSG mice stably engrafted with cells derived from a patient with primary B-ALL leukemia (Fig. 5a). Untreated mice and those treated with control MART-1 (DMF5)^20^ or T3 TCR-transduced T cells showed a high and comparable leukemia burden in BM at baseline (untreated, mean 9.7 ± 3.4%; DMF5, mean 15.6 ± 5.7%; T3, mean 15.4 ± 7.2%), representing a stringent condition for testing of therapeutic efficacy (Fig. 5d). In a clinical setting TdT TCRs will most probably be introduced into autologous T cells^1,2^. Here, we could not obtain sufficient numbers of T cells from the patient sample and we therefore utilized T cells matched for HLA-A2 expression but otherwise not HLA-matched with ALL. Infused T cells contained mainly naïve and central memory T cells and, on average, 34% CD4^+^ T cells (Extended Data Fig. 1c), resembling a clinical product facilitating antitumor reactivity in adoptive T-cell therapy^21,22^. However, a high fraction of naïve T cells also drives alloreactivity^23,24^, as does the presence of a high burden of leukemic cells partially HLA mismatched with T cells. We therefore monitored mice for the likely development of a graft-versus-leukemic effect by comparing leukemic engraftment in the blood of DMF5 (control TCR)-treated relative to untreated mice (Fig. 5b). Detailed end-stage analysis was performed 11 days after T-cell treatment, before the results would be confounded by alloreactivity. At this experimental endpoint, leukemia burden in control mouse groups was very high and comparable in the BM (untreated, mean 42.9 ± 3.4%; DMF5, mean 40.1 ± 4.5%) whereas it was reduced to as little as 0.009 ± 0.005% following treatment with T3 cells (Fig. 5c–e). Leukemia burden in PB and spleen in untreated and DMF5-cell-treated mice was lower than in BM. Nevertheless, a similar and almost complete elimination of leukemic cells was observed in mice treated with T3 cells (PB, mean 0.003 ± 0.001%; spleen, mean 0.002 ± 0.001%) (Fig. 5d). Taken together, leukemic burden was reduced to a level considered negative for MRD (<0.1%) according to criteria set by the Nordic Society of Paediatric Haematology and Oncology (NOPHO) 2008 protocol (no. NCT00816049). TCR-transduced T cells were detected in the BM, PB and spleen of all treated mice (Fig. 5f), with similar numbers of total mononuclear cells (MNCs) between groups (Fig. 5g). Mouse hematopoiesis, which is suppressed in the BM of individuals with leukemic engraftment, was significantly higher in T3 than in DMF5-treated mice (Fig. 5h).

**Fig. 6:**
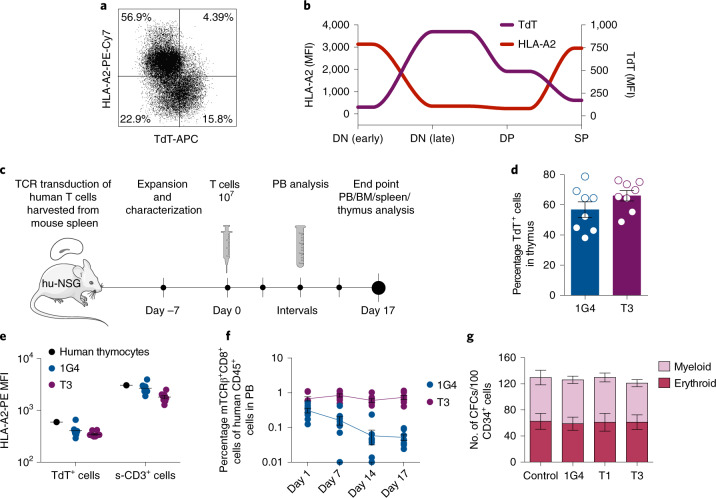
T3 cells preserve healthy hematopoiesis in vivo. **a**,**b**, FACS analysis of viable human CD19^−^HLA-DR^neg^ thymocytes (**a**), and spaghetti-plot illustrating HLA-A2 and TdT mean fluorescence intensity (MFI) at distinct stages of T-cell development (**b**), defined as shown in Extended Data Fig. 9a, in human thymus. **c**, Experimental overview of humanized NSG (hu-NSG) mouse model used to investigate the effect of T3-cell therapy on healthy human hematopoiesis. **d**, Percentage of human TdT^pos^ cells in thymus of hu-NSG mice (*n* = 8 per group). **e**, HLA-A2 mean fluorescence intensity of TdT^pos^ cells (hCD45^+^ TdT^pos^) and surface CD3^+^ (s-CD3^+^) human thymocytes derived from one normal human thymus and from thymus of 1G4 (*n* = 8) and T3 (*n* = 8)-treated humanized mice. **f**, Percentage of TCR-transduced CD8^+^ cells in PB at indicated days after T-cell infusion (*n* = 8 per group). **g**, Myeloid and erythroid colonies generated from sorted normal adult human BM CD34^+^ cells (*n* = 4 biological replicates, data pooled from two independent experiments) following coculture with or without 1G4, T1 or T3 cells for 72 h at an E/T ratio of 2/1. All data are presented as mean ± s.e.m.; dots in **d**–**f** represent individual mice at terminal analysis on day 17 post treatment, unless otherwise stated. Source data

**Extended Data Fig. 9:**
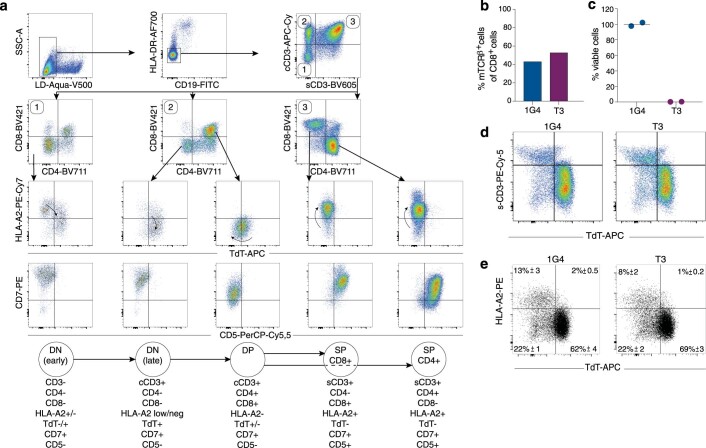
Expression of TdT and HLA-A2 during T cell development. (a) Flow cytometric analysis of thymocytes from normal human thymus removed from a four months old child with congenital cardiac defect (but otherwise healthy) in conjunction with cardiac surgery. Flow plots show gating strategy to define four key developmental stages during thymocyte differentiation, including 1) early double negative (DN), 2) late double negative, 3) double positive (DP) and 4) single positive (SP, either CD4^+^ or CD8^+^) thymocytes. Arrows within the FACS plots in third row indicate direction of differentiation with regard to expression of TdT and HLA-A2 during these developmental stages, illustrated with continuous lines in Fig. 6b. (b) Percentage of TCR-transduced cells among CD8^+^ T cells isolated from spleens of three humanized NSG mice engrafted with healthy cord blood following transduction and expansion, prior to infusion into littermates day 0, as illustrated in Fig. 6c. (c) Functionality of human TCR-transduced T cells shown in (b), as determined by the number of viable BV173 cells present after 72 h co-culture, in percent of corresponding numbers following treatment with 1G4 cells, quantified by flow cytometry (E/T ratio of 1/1). Co-cultures were started on the same day as T cells were injected into mice. Data points show 2 technical replicates from one experiment. (d,e) Representative FACS plots of viable single hCD45^+^CD19^−^CD33^−^ thymocytes from 1G4 or T3-treated humanized NSG mice at terminal analysis on day 17 post T cell infusion illustrating surface CD3 and TdT expression (d) and HLA-A2 and TdT expression (e). Inset numbers show mean ± s.e.m., *n* = 8 per group. Source data

**Extended Data Fig. 10:**
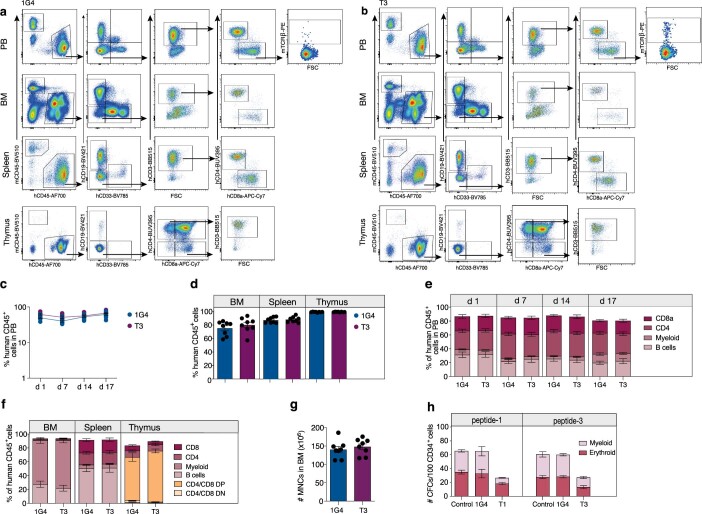
TdT TCR-transduced cells do not impact normal hematopoiesis in vitro or in vivo. (a,b) Representative FACS plots from terminal analysis of viable single MNCs in PB, BM, spleen and thymus from humanized NSG mice treated with 1G4 (a) and T3 (b) cells, to determine the percentage of TCR-transduced T cells and lineage distribution (myeloid: CD33^+^, B cells: CD19^+^, T cells: CD3^+^CD4^+^ or CD3^+^CD8^+^). (c,d) Percentage human CD45^+^ cells (out of human and mouse CD45^+^ cells) in PB at indicated days after T cell infusion (c), and in BM, spleen and thymus of humanized NSG mice treated with 1G4 or T3 cells at terminal analysis on day 17 (d). (e,f) Percentage lineage distribution within human CD45^+^ engrafted cells in PB at indicated days after T cell infusion (e) and in BM, spleen and thymus at terminal analysis (f). (g) BM cellularity at terminal analysis. *n* = 8 per group in c-g. (h) Myeloid and erythroid colonies generated from sorted normal human BM CD34^+^Lineage^−^ cells from one donor co-cultured without T cells (control), or with 1G4, T1 or T3 cells for 48 h in the presence of peptide-1 or peptide-3 at an E/T ratio of 2/1 (2 technical replicates per group). Data are presented as mean ± s.e.m. Source data

Normal peripheral B- and T-cell repertoires are TdT negative and were not affected by T3 or T1 TCRs in in vitro killing assays or during expansion of transduced cells (Fig. 4b and Extended Data Fig. 1b). Studies in which the homeostasis of naïve T cells in normal individuals was measured using retrospective ^14^C dating^25^ support the view that the naïve T-cell repertoire would be sufficiently diverse to sustain peripheral adaptive T-cell immunity for life also in young individuals, and that potential toxic effects on thymocytes would therefore not be a major concern even during prolonged therapy. Thymic contribution to the peripheral repertoire is, however, higher in individuals <20 years of age^26^, raising the possibility that toxicity could be higher in this age group. To address this question, we studied the expression of TdT and HLA-A2 during human thymocyte differentiation. Interestingly, we found that HLA-A2 expression was high only on TdT^neg^ thymocytes, including early double-negative (DN) and single-positive (SP) cells, whereas HLA-A2 expression was downregulated concomitantly with upregulation of TdT on late DN cells transitioning to double-positive (DP) cells (Fig. 6a,b and Extended Data Fig. 9a). We next explored the effect of T3 cell therapy on thymocytes in humanized CD34^+^ NSG mice injected with T3- or 1G4-transduced autologous human T cells harvested from mouse spleen (Fig. 6c). T3 cells were demonstrated to effectively kill a TdT^pos^ cell line in vitro before injection (Extended Data Fig. 9b,c). At the end of the experiment, the fraction of TdT^pos^ thymocytes was similar in T3- and 1G4-treated mice. HLA-A2 levels were similarly low on TdT^pos^ thymocytes compared to surface CD3^+^ TdT^neg^ thymocytes in both experimental groups (Fig. 6d,e and Extended Data Fig. 9d). Correspondingly, only a minor fraction of human thymocytes, replenished by cord blood progenitors in the mouse thymus, were TdT^high^ and HLA-A2^high^ in both groups, as was also observed for thymocytes derived from human thymus (Fig. 6a and Extended Data Fig. 9e). The experiment was ended on day 17 after T-cell injections when TCR-transduced T cells were still detectable in PB, with the fraction of 1G4-transduced T cells declining faster than T3-transduced T cells in PB, possibly suggesting low-level antigen stimulation of T3 T cells (Fig. 6f and Extended Data Fig. 10a,b). No difference between 1G4 and T3 groups was observed following analysis of the distribution of human myeloid and lymphoid lineages in PB, BM, spleen and thymus, as well as cellularity in BM (Extended Data Fig. 10a–g). Further supporting lack of toxicity on hematopoietic stem cells and myeloid progenitor cells, the formation of myeloid and erythroid colonies from adult CD34^+^ BM progenitor cells was not negatively affected following coculture with T1 or T3 cells (Fig. 6g), unless coculture was performed in the presence of peptide-1 or -3 (Extended Data Fig. 10h).

## Discussion

Our study shows that T cells transduced with TCRs recognizing peptides from the cancer target TdT in the context of allogeneic HLA-A2 efficiently killed TdT^pos^HLA-A2^pos^ patient-derived lymphoblastic leukemia cells of B- and T-cell origin in vitro. While the killing efficacy mediated by TdT TCRs will depend on expression levels of TdT, it will also be affected by a number of other factors, such as the expression level of the restricting HLA-A*02:01 molecule, the rate of processing of TdT and presentation on HLA as well as the susceptibility of the target cells to T-cell-mediated killing. In light of that, it was reassuring that TdT TCR-expressing T cells were highly efficient at killing a large number of TdT^pos^ ALL cell lines as well as primary ALL cells.

The TdT TCRs also mediated depletion of engrafted leukemia in vivo in three different mouse models of B-ALL. This included a model of primary leukemia with a high pretreatment leukemic burden. We used TCR-transduced T cells containing a high proportion of naïve T cells, which facilitates persistence and antitumor reactivity in adoptive T-cell therapy^21,22^. In a clinical setting TdT TCRs will most probably be introduced into autologous T cells, but patient-derived T cells were not available in sufficient numbers for the primary diagnosis (PDX) in vivo experiment pursued here. Allogeneic T cells (not HLA-matched except for HLA-A2) containing a high fraction of naïve T cells will, however, also promote alloreactivity^23,24^. To assess the in vivo efficacy and specificity of TdT TCRs, mice were therefore euthanized and all relevant tissues analyzed at a time point before alloreactivity would considerably confound the antileukemic effects specifically induced by TdT TCR. However, despite a very high leukemic burden in control TCR mice, leukemic cells were almost completely eliminated in TdT TCR-treated mice at this time. In the two xenograft cell line models we observed no evidence of an alloresponse after 14–21 days (determined by imaging analysis, which may be less sensitive than the flow cytometric assessment of PB used in the PDX model), at which time almost complete elimination of leukemic cells was observed in mice treated with TdT TCRs. These mice were also examined after 2 months, at which time ALL cells remained undetectable in almost all T3-treated mice, providing support for the longer-term efficacy of TdT TCRs. Since untreated and control TCR-treated mice died or had to be euthanized after 2–3 weeks due to leukemia, as expected in aggressive xenograft cell line models, it cannot be ruled out that alloreactivity in part contributes to the long-term effects in such models. In the future, longer follow-up could be studied in PDX models in which TCR-transduced T cells are derived from the same patient. For T-ALL, in vivo activity of TdT TCRs remains to be tested.

Our data indicate that TCRs could be used in therapy without detrimental toxicity. First, normal T cells, B cells and nonlineage-committed CD34^+^ cells were selectively spared in samples from patients with B- and T-ALL. Second, treatment of humanized mice engrafted with CD34^+^ cord blood cells with autologous human TdT TCR-transduced T cells did not reveal a negative impact on normal hematopoiesis—most importantly, myelopoiesis or T-cell development. These data are supported by in vitro results showing (1) lack of toxicity on myelopoiesis in colony-forming assays and (2) that the majority of thymocytes are TdT^neg^ and that HLA-A2 is downregulated concomitantly with upregulation of TdT, with only a minority of thymocytes expressing both at high levels.

Specificity for TdT peptides and HLA-A2-restriction was confirmed by loss of reactivity to TdT^pos^ leukemia cells in which TdT was knocked out or HLA-A2 was absent, and cognate peptides presented on HLA of primary leukemia cells were directly identified by MS. A variety of HLA-A2^pos^ cell lines that were TdT^neg^ were not recognized, and mapping of TCR reactivity did not identify any naturally occurring 9- or 11-mer peptide in the human proteome that matched the combinations of substituted peptides to which T1 or T3 were reactive. Taken together, the data indicating high efficacy and specificity of TdT TCRs, combined with the unique expression profile of TdT, open new possibilities for the treatment of TdT^pos^ acute leukemia, including patient groups that currently have a dismal prognosis. While our in vitro and in vivo analyses provided no evidence for a major negative impact of TdT TCRs on thymopoesis, the first clinical testing may preferentially focus on adolescent or adult patients in whom thymic output is expected to be modest or very low^25^.

In spite of the success of CAR T-cell therapies in B-cell malignancies, there are currently few options for the 40–50% of patients that relapse^2,5^, and B-ALL patients >25 years are currently ineligible for commercially available CAR 19 T cell therapy due to toxicity concerns. Patients with T-ALL that either have refractory disease, a relapse after allogeneic stem-cell transplantation or very early after first-line therapy, or MRD after relapse induction and consolidation chemotherapy, also have a very poor prognosis. This has prompted the development of CARs targeting pan-T-cell markers such as CD7, CD5, CD3 and CD4 (ref. ^27^). Although fratricide-resistant CAR T cells lacking targets can be generated by genetic engineering^28,29^, the toxicities related to prolonged T-cell depletion might prove unacceptable in the clinic. CAR T cells targeting CD1a^30^, not expressed on mature T cells, might prove attractive for a smaller subset of patients suffering from cortical T-ALL, but that does not include other T-ALL subtypes with higher risk and worse prognosis^10,31^. A CAR targeting the TCR-β chain constant-domain 1, that leaves normal T cells expressing TRBC2 unaffected, is currently under test in a clinical trial including mature T-cell non-Hodgkin lymphoma (no. NCT03590574). However, this CAR is not suitable for T-ALL because only a minority of T-ALL cases have surface expression of TCR^27^.

Off-target toxicity is a serious concern regarding therapeutic TCRs. This risk has proven particularly high for affinity-enhanced TCRs, in some cases causing lethal cross-reactivity in heart and brain^32,33^, and might be related to the fact that such TCRs have not undergone negative thymic selection against any HLA molecule. Due to the inherent degeneracy of TCRs, monospecificity is probably impossible^34^. Nevertheless, recent clinical studies have provided proof of concept for safe and efficacious TCRs^19,35,36^. The goal is thus to identify TCRs that do not show clinically important cross-reactivity. In contrast to affinity-enhanced TCRs, allorestricted TCRs have been negatively selected on up to 12 different HLA alleles. This depletion is substantial, as sequence comparison between, for example, HLA-A alleles shows that TCR contact residues are highly conserved^37^. The highly selective killing mediated by the TdT-specific TCRs T1 and T3 refutes the idea that allorecognition is inherently more cross-reactive than conventional TCR recognition, and the data are supported by structural analysis of allogeneic TCR–pMHC complexes^38,39^. Pioneering studies by Sadovnikova and Stauss^40^, and later by us^41,42^ and others^43^, suggest that allorestricted TCRs can be utilized in cancer immunotherapy. However, the dogma that allo-HLA-restricted TCRs are inherently cross-reactive and mainly recognize the HLA molecule itself has prevailed, limiting therapeutic utility. The question has thus remained whether the few identified allo-HLA-restricted TCRs that seemed to be peptide specific represent rare exceptions, or whether peptide specificity has not been sufficiently investigated. Recent advances in technologies for mapping of TCR reactivity allow for highly sensitive identification of peptides to which a candidate therapeutic TCR might be cross-reactive. Screening of TCR reactivity to unbiased peptide libraries, including yeast two-hybrid assays^44^ and genome-wide libraries introduced into target cells by lentivirus^45^, demonstrated that the tested TCRs cross-reacted to only those peptides harboring an amino acid sequence partly shared with the cognate peptide. Moreover, studies investigating binding of peptide–MHC tetramers with single versus multiple simultaneous amino acid substitutions showed that bioinformatic searches for combinations of hits for single amino acid substitutions, such as that performed for TdT TCRs, were sufficient to identify candidate cross-reactive peptides^46^.

Cell-type-specific antigens are often, unlike cancer testis antigens^47^, homogeneously expressed in malignant cells. Targeting of broadly expressed lineage markers may, however, be prohibitively toxic. The identification of TCRs that efficiently and selectively recognize TdT paves the way for targeting of cell-type-specific, intracellular antigens that are transiently expressed during differentiation. TdT-specific TCRs could represent an attractive therapeutic option for patients with B-ALL ineligible for, or relapsing from, CD19-specific CAR T-cell therapy, or for patients with T-ALL relapsing from chemotherapy or allogeneic stem-cell transplantation and for whom no cellular immunotherapy currently exists. If successful, the patient groups that might benefit from such T-cell therapy could be expanded.

## Methods

This study was approved by the Regional Committee for Medical and Health Research Ethics (REC) South-East, Norway (nos. 2018/879, 2018/1246, 2019/31516), the Institutional Review Board and the Data Protection Officer, Oslo University Hospital, Swedish Ethical Review Authority, Stockholm (no. EPN 2018/901-31) and was performed in accordance with the Declaration of Helsinki.

### Primary patient cells, healthy blood donor cells and cell lines

Pediatric and young adult relapsed/refractory patients with B-ALL were enrolled into and treated according to CAR T-cell trials ClinicalTrials.gov Identifier nos. NCT02435849 and NCT03123939, and pediatric patients with T-ALL according to NOPHO-ALL-2008 (no. NCT00816049). Institutional review board and REC approvals for the use of primary human diagnostic blood and BM samples from pediatric and adult patients in the study were obtained, as was informed written consent from patients or their guardians. Thymocytes were isolated from human thymus removed as a consequence of routine procedures for open cardiac surgery to correct a congenital cardiac defects (in an otherwise healthy child) following informed written consent from guardians (ethical approval no. 2019/31516). PB MNCs (PBMCs) from healthy donor buffy coats were obtained from the blood bank of Oslo University Hospital, and PB or BM MNCs from leukemia patients were obtained from biobanked, cryopreserved material (ethical approval nos. 2018/879 and 2018/1246). MNCs were isolated by density-gradient centrifugation (Axis-Shield) and were typed for HLA-A2 expression by flow cytometry. EBV-LCLs were generated from HLA-A2^pos^ and HLA-A2^neg^ PBMCs as described previously^48^. Thymic tissue was cleaned from blood clots, connective tissue, fat tissue and necrotic tissue and cut into small pieces that were gently triturated with a 1-l micropipette attached to a wide-bore pipette tip to release thymocytes into cold RPMI-1640 medium. Thymocytes were washed twice in medium and then cryopreserved.

The following cell lines were used in the study: NALM-6, BV173, EBV-LCL, HPB-ALL, T2, REH, RD, U-2 OS, FM-6, HeLa, HaCaT, COLO688, EA.hy926, U-87 MG, Daoy, HCT-116, CHP-212, MCF7, K562, RS4;11 and Phoenix-AMPHO. Cell lines were obtained from the American Tissue Culture Collection and Deutsche Sammlung von Mikroorganismen und Zellkulturen, and cryopreserved aliquots were labeled according to passage. Only low passages (one to four) were used to start cultures. The identity of cell lines with high passages (five or higher) used experimentally was ascertained by short tandem repeat DNA profiling, a service provided by Labcorp DNA Identification Lab (formerly Genetica, https://celllineauthentication.com/). Cell lines were cultured in humidified cell incubators containing 5% CO_2_ at 37 °C in media as instructed by the supplier, and regularly tested for mycoplasma contamination.

### Induction of antigen-specific T cells

Induction of T cells reactive to TdT peptides and generation of cytotoxic T-cell lines and clones were performed as previously described^16^, with some modifications. Briefly, monocytes were isolated from PBMCs of HLA-A2^pos^ healthy donors on day 4 using CD14-reactive microbeads and AutoMACS Pro Separator (Miltenyi Biotec), and cultured for 3 days in CellGro GMP DC medium (CellGenix) supplemented with 1% (v/v) human serum (HS, Trina biotech), 1% (v/v) penicillin/streptomycin, 50 IU ml^–1^ interleukin (IL)-4 (PeproTech) and 800 IU ml^–1^ GM-CSF (Genzyme). Subsequently MoDCs were matured for 14–16 h by the addition of lipopolysaccharide (Sigma-Aldrich) and IFN-γ (PeproTech) to final concentrations of 10 ng ml^–1^ and 100 IU ml^–1^, respectively. On day −1, naïve CD8^+^ T cells were isolated from PBMCs from HLA-A2^neg^ donors by the AutoMACS Pro Separator and CD8^+^ T-cell isolation kit premixed with CD45RO- and CD57-reactive beads (Miltenyi Biotec). On day 0, MoDCs were harvested, electroporated with mRNA encoding full-length TdT and cocultured with naïve T cells in DC-T cell medium supplemented with 30 ng ml^–1^ IL-21 (PeproTech) at a DC/T-cell ratio of 1/4. Parallel control cocultures were initiated with MoDCs transfected with irrelevant mRNA. Between days 8 and 10, cocultures were screened for the presence of TdT pMHC multimer-reactive CD8^+^ T cells. pMHC multimers labeled with phycoerythrin (PE) and allophycocyanin (APC) were prepared in-house as described previously^49,50^. Live CD8^+^ T cells staining positively for PE- and APC-conjugated pMHC multimers were sorted subsequently by fluorescence activated cell sorting (FACS).

### Sorting and cloning of pMHC multimer^+^ CD8^+^ T cells

Single-cell cloning of peptide-1 and peptide-3 MHC multimer^+^ CD8^+^ T cells was performed as described previously^16^. Briefly, PBMCs from three different donors were mixed in 1/1/1 ratio, irradiated with 35 Gy, washed and resuspended in X-vivo 20 medium (Lonza, BioNordika) supplemented with 5% (v/v) human serum and 1% (v/v) penicillin/streptomycin (T-cell medium). Feeder cells were added to 96-well tissue-culture-treated plates (0.2 × 10^6^ cells per well in a volume of 100 μl) and 100 μl of T-cell medium containing 2 μg ml^–1^ phytohaemagglutinin (Remel Thermo Scientific), 80 ng ml^–1^ IL-2 (R&D Systems), and 4 ng ml^–1^ IL-15 (PeproTech) was added to the feeder cells. MoDC/T-cell cocultures were harvested and stained with anti-CD3 and -CD8a antibodies, and PE- and APC-conjugated pMHC multimers. CD8^+^ and double-pMHC multimer^+^ cells were sorted as single cells into 96-well plates containing feeder cells using either FACS Aria II (BD Biosciences) or a SH800 cell sorter (Sony Biotechnology). Every 7 days, cultures were supplied with fresh T-cell medium containing IL-2 and IL-15 and expanding clones were identified by microscopy. On day 14 after FACS, growing clones were restimulated with feeder cells prepared as described above. T-cell clones were stained with relevant pMHC multimers. As negative controls, peptide-1 reactive clones were stained with peptide-3 MHC multimers and vice versa. To assess functionality, T-cell clones were stimulated with EBV-LCLs pulsed with relevant peptides and the NALM-6 cell line naturally expressing TdT, and assessed for upregulation of CD137.

### TCR sequencing and cloning

Paired TCR-α and -β chains from three clones reactive to peptide-1 and one reactive to peptide-3 were amplified using a protocol described previously, which was modified and adapted for the targeted amplification of TCRα and β transcripts^51,52^. In brief, RNA was extracted and processed to obtain TCR-specific complementary DNA. Four pairs of TCR-α/β constant-domain-specific primers were used to run PCR with reverse transcription for each clone, followed by the addition of a poly-G tail and template switch to obtain double-stranded DNA. Finally, two rounds of nested PCR amplification were performed using additional constant-domain primers and adapter primers annealing to an anchor sequence introduced in the poly-G domain. A Kappa Illumina kit was utilized to prepare libraries, which were later sequenced on an Illumina MiSeq. MiTCR script was used to analyze sequencing data, and an in-house python script TCRprimer was used to reconstruct full-length TCR chains as described previously^51,53^. Output was manually verified for each sample in IMGT/V-Quest^54^. Variable TCR-α and -β fragments of identified TCRs were codon-optimized, synthesized and cloned by Genscript.

### Gene transfer to human PBMCs and cell lines

T1 and T3 TCRs were transduced into HLA-A2^pos^ donor-derived and patient-derived PBMCs. 1G4 and DMF5 TCRs were also transduced into HLA-A2^pos^ donor-derived PBMCs as controls for in vivo experiments. For stimulation of human PBMCs, 6- or 12-well tissue-culture-treated plates were coated with anti-CD3 (clone OKT3, eBioscience) and anti-CD28 (clone CD28.6, eBioscience) antibodies. PBMCs (2 × 10^6^ cells ml^–1^) in T-cell medium supplemented with IL-7 and IL-15 (5 ng ml^–1^ each, PeproTech) were added to antibody-coated plates and incubated at 37 °C and 5% CO_2_ for 72 h. For generation of retroviral supernatants, 4 × 10^6^ Phoenix-AMPHO packaging cells were plated in 10-cm Petri dishes for 24 h, and cells were transfected with γ-retroviral vector DNA and mixed in X-tremeGENE 9 DNA Transfection reagent (Roche Diagnostics) and Opti-MEM. The following day, medium was refreshed and cells were incubated at 32 °C and 5% CO_2_ for 24 h. Subsequently, PBMCs were harvested, resuspended in T-cell medium supplemented with IL-7 and IL-15, mixed with retroviral supernatant, placed in non-tissue-culture-treated 6-well plates precoated with Retronectin (20 μg ml^–1^, Takara) and spinoculated at 900*g* for 60 min. A second spinoculation was performed the following day with fresh retroviral supernatant, and transduction efficiency was determined after 3–5 days by staining with anti-mouse TCR-β chain antibody and/or pMHC multimer followed by flow cytometry. Before functional experiments, cells were cultured for 48–72 h in T-cell medium containing low concentrations of cytokines (0.5 ng ml^–1^ IL-7 and IL-15). Alternatively, cells were frozen for later experiments.

Retroviral supernatants containing viral DNA encoding full-length HLA-A2 and TdT were also produced as described above and utilized to transduce REH, RD, HeLa, K562, HaCaT, COLO688, EA.hy926 and HPB-ALL cell lines with HLA-A2, and EBV-LCL with TdT. For in vivo experiments, the BV173 and NALM-6 cell lines were stably transduced to express firefly luciferase and GFP, and were designated as BV173^*ffluc-eGFP*^ and NALM-6^*ffluc-eGFP*^. All transduced cell lines were subsequently purified by FACS sorting, expanded and frozen for use in later experiments.

Complementary DNA for TdT and HLA-A2 was cloned into the pCIpA102 vector for mRNA production, as previously described^42^

### Antibodies and flow cytometry

Flow cytometry was performed on either a BD LSR II flow cytometer (BD Biosciences), FACSCanto II (BD Biosciences), FACS Aria Fusion (BD Biosciences) or MACSQuant (Miltenyi Biotec), and data were analyzed using FlowJo (TreeStar) or FACS DIVA (BD Biosciences) software. For surface antibody staining, antibodies were added to cells for 15–20 min on ice followed by washing steps. For intracellular staining, cells were suspended in Cytofix/Cytoperm (BD Bioscience) solution for 20 min, washed with Perm/Wash buffer (BD Bioscience) and then stained with antibodies. The following fluorescently conjugated anti-human antibodies were acquired from BD Biosciences or BioLegend, unless otherwise specified: anti-CD14 (no. HCD14, 61D3, eBioscience), -HLA-A2 (no. BB7.2), -CD62L (no. DREG-56), CD56 (no. HCD56, B159), -CD57 (no. HNK-1), -CD45RO (no. UCHL1), -CD45RA (no. HI100), -CCR7 (no. 150503), -CD137 (no. 4B4-1), -CD45 (no. HI30), -TdT (no. E17-1519), -CD10 (no. HI10a), -CD19 (no. HIB19, SJ25C1), -CD38 (no. HIT2), -CD34 (no. 581, 8G12), -CD1a (no. HI149), -CD2 (no. S5.2, RPA-2.10), -CD3 (no. UCHT1, OKT3, HIT3a), -CD8a (no. RPA-T8), -CD4 (no. RPA-T4), -CD5 (no. UCHT2, L17F12), -CD7 (no. M-T701), -CD33 (no. WM-53), -CD11b (no. ICRF44), -CD20 (no. 2H7), -CD235a,b (no. HIR2) anti-mouse CD45 (no. 30-F11), -CD45.1 (no. A20), -CD99 (no. DN16, Bio Rad), Ter-119 (no. TER-119) and -CD3 (no. SK7, eBioscience). Anti-mouse TCR-β chain PE (no. H57-597, BD Biosciences) was used to test the transduction efficiency of T1 and T3 TCRs in human cells, and to monitor transduced T cells used for in vivo treatment in mice. Live/Dead Fixable Near-IR Dead Cell Stain kit (Life Technologies), Live/Dead Fixable Aqua Dead Cell Stain kit (Life Technologies), DAPI (Invitrogen) or 7-AAD (BioLegend) was used to exclude dead cells in flow-cytometry experiments. A list of antibodies used for flow cytometry or ELISA is provided in Supplementary Table 5, including information about supplier, species, clone name, fluorochromes, dilution factor used and application. Unconjugated anti-HLA class I antibody (no. W6/32, BioLegend), at a concentration of 20 μg ml^–1^, was used to block MHC class I on REH and HPB-ALL cell lines transduced with HLA-A2.

### T-cell activation assays

The reactivity of T-cell clones and TCR-transduced T cells was investigated by measurement of CD137 upregulation or IFN-γ release. Briefly, 100,000 cells per well of indicated target cell lines or primary patient tumor cells were coincubated with T-cell clones or TCR-transduced PBMC (50,000 cells per well). Where indicated, target cells were pulsed with the specified concentrations of peptide for 1–2 h or electroporated with mRNA encoding full-length TdT, washed and then cocultured with effector cells. Following 14–16 h of coincubation, plates were centrifuged at 400*g* for 3 min. Culture supernatants were harvested for measurement of IFN-γ by ELISA, and remaining cells were stained for flow cytometry to measure upregulation of CD137 on live CD8^+^ T cells. Results are reported either as percentage of CD137^+^ of CD8^+^ cells (labeled as CD137^+^/CD8^+^ cells) or percentage of CD137^+^ events among TCR-transduced CD8^+^ T cells (only for TCR-transduced patient T cells in Extended Data Fig. 8). In some experiments, cells were labeled with 0.75 μM of the fluorescent cell staining dye CellTrace Violet (CTV, Life Technologies) or carboxyfluorescein succinimidyl ester (CFSE, Life Technologies) to distinguish between target and effector cells. The following reagents were acquired from BD Pharmingen or R&D Systems: mouse anti-human IFN-γ capture antibody (no. NIB42), Biotin Mouse Anti-Human IFN-γ detection antibody (no. 4 S.B3), streptavidin-HRP, stabilized tetramethylbenzidine and hydrogen peroxide as substrate solutions, sulfuric acid as stop solution and recombinant human IFN-γ protein as standard. Assays were performed according to the manufacturers’ instructions. Levels of IFN-γ in serum harvested from mouse PB on day 2 after T-cell therapy were measured by Multiplexed Bead-Based Cytokine Assay from BD Pharmingen—the Human Th1/Th2/Th17 CBA Kit. The assay was preformed according to the manufacturer’s instructions.

### Proliferation assay

For the proliferation assay (Extended Data Fig. 4), 100,000 target cells were cocultured with 1G4, T1 or T3 TCR-transduced PBMCs for 5 days in T-cell medium at a 1/1 ratio in a round-bottom, 96-well plate in triplicate. To observe the presence or absence of proliferation for both CD4^+^ and CD8^+^, transduced cells were labeled with 0.75 µM of CSFE (Life Technologies). Following coculture, cells were harvested, washed and stained with human anti-CD3, -CD4, -CD8, -CD19, -CD10 and Live/Dead Fixable Near-IR to exclude dead cells. After 15 min, cells were washed and resuspended in 200 μl of FACS buffer containing 10,000 CountBright Absolute Counting Beads (ThermoFisher). Following data acquisition, an equal number of bead events (3,000) was recorded from every well. Data are shown as histograms displaying CFSE levels for both CD4^+^ and CD8^+^ populations among CD3^+^, CD19^−^, CD10^−^ and Live/Dead Fixable Near-IR negative events, and as a percentage of the mean of live proliferating CD4^+^ and CD8^+^ T cells acquired from three parallel wells.

### Flow cytometry-based cytotoxicity assay using cell lines as targets

For cytotoxicity assays on B- and T-ALL cell lines, 50,000 target cells in T-cell medium were cocultured for 48 h with T1 or T3 TCR-transduced PBMCs in a round-bottom, 96-well plate in triplicate. Effector cells were defined as TCR-transduced CD8^+^ T cells in the PBMC product (routinely >90% transduction efficiency and 55–65% CD8^+^ T cells, the remainder being CD4^+^ T cells), with an E/T ratio of 1/1. Following coculture, cells were harvested, washed and stained with human anti-CD3, -CD8, -CD19 and Live/Dead Fixable Near-IR to exclude dead cells. After 15 min, cells were washed and resuspended in 200 μl of FACS buffer containing 10,000 CountBright Absolute Counting Beads (ThermoFisher). Following data acquisition, an equal number of bead events (5,000) was recorded from every well. Data were normalized and reported as a percentage of the mean of live tumor cell numbers acquired from three parallel wells cocultured with mock-transduced T cells from the same donor. An example of the gating strategy used to identify live tumor cells is shown in Extended Data Fig. 4f.

### Flow cytometry-based assays for T-cell activation and cytotoxicity using primary human B- and T-ALL samples

Peripheral blood or BM samples from patients with B- and T-ALL were thawed and resuspended in T-cell medium containing low concentrations of IL-7 and IL-15 (0.5 ng ml^–1^). Cells were transferred to round-bottom, 96-well plates for assays measuring CD137 upregulation on TCR-transduced T cells or cytotoxicity on target cells. Individualized antibody panels and gating strategies used to identify malignant blasts and normal leukocyte populations were designed after review of diagnostic phenotyping available in hospital records. Allogeneic or, for patient no. 1N, autologous patient-derived, T cells transduced with TCRs were used in experiments. TCR-transduced cells were prelabeled with CTV dye to distinguish them from target cells. Wherever indicated, target cells were loaded with relevant peptides for 1–2 h, washed and then coincubated with TCR-transduced T cells for measurement of CD137 upregulation, as described above. For cytotoxicity assays, 50,000 target cells per well were coincubated with equal numbers of effector cells in two to four parallels per condition for 48–72 h and then stained with individualized antibody panels for flow cytometry. CountBright Absolute Counting Beads were utilized for acquisition of standardization, and data were normalized and reported as described above. For visual display of flow-cytometry plots, we utilized unsupervised nonlinear dimensionality reduction algorithms such as *t*-SNE using FlowJo (TreeStar) software.

### Knockout of TdT in NALM-6 cells

CRISPR–Cas9-mediated knockout was done as previously described^55^ using the guide RNA 5’-ggc gct atg cca cac atg ag-3’ to target the TdT epitope located at the end of exon 10 of the TdT gene. Following electroporation, modified NALM-6 cells were enriched by FACS to generate a bulk culture showing heterogeneous TdT knockout (NALM-6-ΔTdT-bulk). A clone was generated by limited dilution that showed the same partial deletion of the target epitope (NALM-6-ΔTdT_475–481_) in both alleles. To verify TdT knockout, genomic DNA was isolated from indicated cell lines (GeneJET Genomic DNA Purification Kit, ThermoFisher) and the targeted region was amplified by PCR (Phusion, ThermoFisher: sense, 5’-tca cta gag gga tgt agc cac c-3’; antisense, 5’-act cat tgc caa cac caa gg-3’), then PCR fragments were purified (Invisorb Fragment CleanUp, Stratec) and sent for sequencing (Eurofins Genomics) using the indicated PCR primers.

### In vivo TdT TCR T-cell activity in two xenograft B-ALL cell line models

This work was approved by the Norwegian Food Safety Authority (application ID: 17500). All experiments were performed in compliance with the institutional guidelines and 2010/63/EU directive on the protection of animals used for scientific purposes. Male and female NOD-*scid* IL2Rg^null^ (NSG) mice, 8–10 weeks old and bred in-house were used in these experiments. On day 11, mice were sublethally irradiated with 2.5-Gy radiation using a MultiRad225 X-ray irradiator (RPS services). Next, 4 × 10^6^ or 1 × 10^6^ cells of human B-ALL cell lines BV173 or NALM-6, retrovirally transduced to express GFP and firefly luciferase, were injected on day 10 through the tail vein. After leukemia was established and confirmed by BLI on day –1, mice were treated with 10^7^ PBMC transduced with either T1, T3 or a control TCR targeting NY-ESO-1 (1G4)^19^. A separate group of control mice did not receive any T-cell injections. To ensure T-cell survival, mice were injected intraperitoneally daily with 2,500 IU IL-2 (R&D Systems) followed by BLI (IVIS Spectrum in vivo imaging system, and analysis by Living image software v.4.5.2, PerkinElmer), and blood analysis was performed by flow cytometry at different intervals. For survival analysis, mice were observed for clinical signs of tumor spread and were sacrificed if they developed >20% weight loss, hunched posture, ruffled fur or limb paralysis. Experiments were terminated 2 months after T-cell injection to avoid graft-versus-host disease, and surviving mice in treated groups were humanely sacrificed. In two experimental cohorts (BV173 or NALM-6), BM from surviving T3-treated mice at the end of experiment (day 57 or 60), or from 1G4-treated or untreated mice sacrificed due to high leukemia burden, was harvested and processed for flow cytometry to determine the presence of T cells and tumor cells, and expression of TdT and HLA-A2.

### In vivo TdT TCR T-cell activity in a patient-derived xenograft model

Experiments were performed according to the guidelines and permissions obtained from the ethics committees at Stockholm Norra Djurförsöksetisks Nämd (no. 17978-2018). Experimental mice were housed at two to five per cage in IVC-Mouse GM 500 cages with a light cycle of 06.00–18.00, 21 °C and 50% humidity. Female NOD.Cg-Prkdc^scid^ Il2rg^tm1Wjl^/SzJ mice (NSG; Jackson Laboratory, no. 005557) 9–15 weeks of age were sublethally irradiated with two doses of 1.65 Gy (X-ray source) 4 h apart. Viable, T-cell-depleted BM cells from HLA-A2^pos^ B-ALL patient no. 20O were yield sorted on a BD FACS Aria Fusion by exclusion of 7-AAD^+^ and CD3^+^ cells, and 4 × 10^5^ cells were injected via the tail vein of NSG mice 4–6 h after the final irradiation dose. Stable engraftment was confirmed by PB analysis and BM aspiration from all transplanted mice 18–19 and 20–26 days after transplantation, respectively. NSG mice were allocated into untreated, DMF5 and T3 T-cell groups based on their engraftment levels so that mean human leukemic engraftment was comparable among groups. Then, 7.5 × 10^6^ mTCR-β^+^CD8^+^ T cells transduced with DMF5 TCR or T3 TCR were injected 22–25 days after transplantation of primary B-ALL cells, and all groups received a daily intraperitoneal injection of 2,500 IU of IL-2 (R&D Systems) per mouse. Engraftment was monitored in PB 3 and 10 days after T-cell infusion. Following euthanization of the mice 11 days after T-cell infusion, BM, spleen and PB were subjected to detailed flow-cytometry analysis to identify leukemic cells and infused T cells. For analysis of BM samples, a minimum of 1.8 × 10^5^ events were acquired for all mice and at least 3 × 10^5^ events were acquired for all but two T3-cell-treated mice to determine MRD levels according to NOPHO guidelines.

Cell counting of BM (two tibiae, two femora and two crista) and spleen from euthanized mice was performed using a Sysmex hematologic cell counter. TrueCount beads (BD Biosciences) were added to whole PB according to the manufacturer’s instructions and stained for mouse CD45.1 and human CD45 to determine absolute MNC counts per microliter of blood. Leukemia burden and TCR-transduced CD8^+^ cells were quantified for each tissue based on the frequency of human CD45^+^CD19^+^CD10^+^ and human CD45^+^CD3^+^CD8^+^mTCR-β^+^ cells, respectively, in relation to total cell count.

### In vivo impact of T3-cell treatment on normal human hematopoiesis in humanized NSG mice

Experiments were performed according to the guidelines and obtained permissions from the ethics committees at Stockholm Norra Djurförsöksetisks Nämd (no. 17978-2018). Experimental mice were housed at two to five per cage in IVC-Mouse GM 500 cages with a light cycle of 04.00–16.00, 21 °C and 50% humidity. Female NSG mice stably engrafted with HLA-A2^pos^ human cord blood cells were purchased from the Jackson Laboratory. Following confirmation of human engraftment 24 weeks after transplantation, single-cell suspensions from spleens of three engrafted NSG mice were transduced with 1G4 or T3 TCR constructs and expanded as described above. Activity of NSG-derived 1G4 and T3 cells was validated in vitro by performing flow-cytometry-based cytotoxicity assay on BV173 cells, in parallel with infusion of 10 × 10^6^ T cells into the remaining humanized NSG mice through tail vein injection. Because engrafted mice also contained endogenous cord-blood-derived T cells with autocrine IL-2 production potential, supportive daily intraperitoneal infusion of 500 IU of IL-2 was not included for half of the mice (since no differences were observed with or without IL-2 supportive infusions, the data from these groups were pooled). The persistence of infused T cells was monitored in PB, and the impact of therapy on mature blood cell lineages was monitored in PB, spleen, thymus and BM by flow cytometry 17 days after T-cell infusion. The impact on human T-cell progenitors in mouse thymus was investigated through surface and intracellular FACS staining. Cell counting of BM (two tibiae, two femora and two crista) from terminated mice was performed using a Sysmex hematologic cell counter.

### In vitro impact on clonogenic potential of normal hematopoietic progenitors

Bone marrow MNCs were obtained from four HLA-A2^pos^ healthy donors collected at Karolinska University Hospital, with informed consent and ethical approval (no. EPN 2018/901-31). Two hundred and fifty viable single CD34^+^ progenitor cells, identified by DAPI and mature lineage exclusion, were sorted on a BD FACS Aria Fusion and cocultured with 500 CD4^−^CD19^−^ (sorted on BD FACS Aria Fusion) 1G4, T1 or T3 TCR-transduced T cells in StemSpan SFEM (StemCell Technologies), supplemented with 10% BIT9500 (StemCell Technologies), penicillin/streptavidin (100 U ml^–1^; Hyclone Laboratories), 2-β-mercaptoethanol (2-ME, 0.1 mM; Sigma-Aldrich), stem cell factor (SCF, 10 ng ml^–1^), flt3 ligand (FL, 10 ng ml^–1^), thrombopoietin (TPO, 10 ng ml^–1^), interleukin-3 (IL-3, 5 ng ml^–1^), granulocyte colony-stimulating factor (G-CSF, 10 ng ml^–1^), granulocyte macrophage colony-stimulating factor (GM-CSF, 10 ng ml^–1^) and erythropoietin (EPO, 1 U ml^–1^) in 37 °C, 5% CO_2_. CD34^+^ lin^−^ progenitors cultured without T cells were used as controls. After 72 h, cells from the cocultures were transferred to cytokine-containing methylcellulose (MethoCult, no. H4434, StemCell Technologies) in Iscove’s modified Dulbecco’s Medium (Gibco) supplemented with 20% fetal bovine serum (Sigma-Aldrich), L-glutamine (2 mM, Sigma-Aldrich), penicillin/streptavidin (100 U ml^–1^) and 2-ME (0.1 mM), to facilitate colony generation. After 14 days in methylcellulose, colonies were scored under an inverted microscope as myeloid or erythroid. As a positive control, CD34^+^ lin^−^ progenitors were externally loaded with 1 μM peptide-1 or peptide-3 in StemSpan SFEM for 2 h, followed by 48 h of coculture with or without transduced T cells in the presence of 100 nM peptides, and colonies were scored 10 days after transfer of cells to methylcellulose as described above.

### Peptide–HLA-A2 model generation

To generate models, a list of existing peptide–HLA-A2 structures from Protein Data Bank (PDB) was compiled. More than ten HLA-A2 structures were solved presenting nonameric peptides. We set out to use a high-resolution model (PDB accession code 5MEQ)^56^ in which HLA-A2 presents a peptide (ILAKFLHTL) from human telomerase reverse transcriptase. For the peptide–3HLA-A2 model, our options were much more limited since considerably fewer HLA-A2 structures were solved presenting undecamer peptides. We used a high-resolution model (PDB accession code 5D9S)^57^ in which HLA-A2 presents an 11-mer peptide (FVLELEPEWTV) derived from *Toxoplasma gondii*. All peptide residues presented by HLA-A2 from these two models were replaced using pymol mutagenesis, and residues with minimum clashes with HLA-A2 were selected for generation of the peptide-1– and peptide-3–HLA-A2 models.

### Statistical analysis

Statistical analysis was performed in GraphPad Prism v.6–8 (GraphPad Software). To compare more than two experimental groups, ordinary one-way analysis of variance (ANOVA) test with adjustment for multiple comparisons with Tukey’s post-test was employed. Survival analysis was performed using log-rank (Mantel–Cox) test. To determine differences between in vivo treatment groups in the PDX and humanized NSG mouse models, Kruskal–Wallis ANOVA by Dunn’s multiple comparisons test and two-tailed Mann–Whitney test were performed. *P* < 0.05 was considered statistically significant.

### Reporting Summary

Further information on research design is available in the Nature Research Reporting Summary linked to this article.

## Online content

Any methods, additional references, Nature Research reporting summaries, source data, extended data, supplementary information, acknowledgements, peer review information; details of author contributions and competing interests; and statements of data and code availability are available at 10.1038/s41587-021-01089-x.

## Supplementary information

Supplementary Information Supplementary Figs. 1 and 2, Tables 1–5 and methods.

Reporting Summary

## Data Availability

The data that support the findings of this study are included in the manuscript and in the Supplementary information. Additional datasets used in the study are: PDB (accession codes: 5MEQ (https://www.rcsb.org/structure/5MEQ) and 5D9S (ttps://www.rcsb.org/structure/5D9S); *Homo sapiens* canonical database, using the Mascot search engine v.2.2.04 (www.matrixscience.com); Uniprot *Homo sapiens* database, using Mascot v.2.2.07 (www.matrixscience.com); curated human proteome databases UniProtKB/Swiss-Prot and PDB by ScanProsite tool (https://prosite.expasy.org/scanprosite/); and peptide–MHC class I binding prediction algorithm NetMHC v.4.0 (http://www.cbs.dtu.dk/services/NetMHC/). Source data are provided with this paper.

## Source data

Source Data Fig. 1 Source data.
Source Data Fig. 2 Source data.
Source Data Fig. 3 Source data.
Source Data Fig. 4 Source data.
Source Data Fig. 5 Source data.
Source Data Fig. 6 Source data.
Source Data Extended Data Fig. 1 Source data.
Source Data Extended Data Fig. 2 Source data.
Source Data Extended Data Fig. 3 Source data.
Source Data Extended Data Fig. 4 Source data.
Source Data Extended Data Fig. 5 Source data.
Source Data Extended Data Fig. 6 Source data.
Source Data Extended Data Fig. 7 Source data.
Source Data Extended Data Fig. 8 Source data.
Source Data Extended Data Fig. 9 Source data.
Source Data Extended Data Fig. 10 Source data.
