# Supplementary material for: T cells targeted to TdT kill leukemic lymphoblasts while sparing normal lymphocytes
Source: Nat Biotechnol. Author manuscript; Available in PMC 2022 Apr 19. (PMC9005346; doi:10.1038/s41587-021-01089-x)
Supplement: Supplementary information [file EMS140577-supplement-Supplementary_information.pdf]

nature portfolio

https://doi.org/10.1038/s41587-021-01089-x

Supplementary information

T cells targeted to TdT kill leukemic lymphoblasts while sparing normal lymphocytes

In the format provided by the
authors and unedited

T cells targeted to TdT kill leukemic lymphoblasts while sparing normal lymphocytes

Muhammad Ali[1,2,*], Eirini Giannakopoulou[1,2,*], Yingqian Li[1,2], Madeleine Lehander[3], Stina Virding Culleton[3], Weiwen Yang[1,2], Cathrine Knetter[1,2], Mete Can Odabasi[4], Ravi Chand Bollineni[1,2], Xinbo Yang[5], Zsofia Foldvari[1,2], Maxi-Lu Böschen[1,2], Eli Taraldsrud[1,2], Erlend Strønen[1,2], Mireille Toebes[6], Amy Hillen[3], Stefania Mazzi[3], Arnoud H. de Ru[7], George M.C. Janssen[7], Arne Kolstad[8,2], Geir Erland Tjønnfjord[9], Benedicte A. Lie[10], Marieke Griffioen[11], Sören Lehmann[3,12], Liv Toril Osnes[13], Jochen Buechner[14], K. Christopher Garcia[5], Ton N. Schumacher[6,15], Peter A. van Veelen[7], Matthias Leisegang[4,16,17,18], Sten Eirik W. Jacobsen[3,19,20,21‡], Petter Woll[3‡], Johanna Olweus[1,2 #]

[1]Department of Cancer Immunology, Institute for Cancer Research, Oslo University Hospital Radiumhospitalet, Oslo, Norway

[2]K.G. Jebsen Center for Cancer Immunotherapy, Institute of Clinical Medicine, University of Oslo, Norway

[3]Department of Medicine Huddinge, Center for Hematology and Regenerative Medicine, Karolinska Institutet, Stockholm, Sweden

[4]Charité – Universitätsmedizin Berlin, Institute of Immunology, Berlin, Germany

[5]Departments of Molecular and Cellular Physiology and Structural Biology, Stanford University School of Medicine, Stanford, CA, USA

[6]Division of Molecular Oncology & Immunology, Oncode Institute, The Netherlands Cancer Institute, Amsterdam, The Netherlands

[7]Center for Proteomics and Metabolomics, Leiden University Medical Center, Leiden, The Netherlands

[8]Department of Oncology, Oslo University Hospital Radiumhospitalet, Oslo, Norway

[9]Department of Haematology, Oslo University Hospital and KG Jebsen Center for B cell malignancies, Institute of Clinical Medicine, University of Oslo, Oslo, Norway

[10]Department of Medical Genetics, University of Oslo and Oslo University Hospital, Oslo, Norway
[11]Department of Hematology, Leiden University Medical Center, Leiden, The Netherlands
[12]Department of Medical Sciences, Uppsala University Hospital, Uppsala, Sweden
[13]Department of Immunology, Oslo University Hospital, Oslo, Norway
[14]Department of Pediatric Hematology and Oncology, Oslo University Hospital, Oslo, Norway
[15]Department of Immunohematology and Bloodtransfusion, Leiden University Medical Center, Leiden, The Netherlands
[16]David and Etta Jonas Center for Cellular Therapy, The University of Chicago, Chicago, IL 60637, USA
[17]German Cancer Consortium (DKTK), partner site Berlin, Berlin, Germany
[18]German Cancer Research Center (DKFZ), Heidelberg, Germany
[19]Department of Cell and Molecular Biology, Karolinska Institutet, Stockholm, Sweden
[20]Karolinska University Hospital, Stockholm, Sweden
[21]MRC Molecular Haematology Unit, MRC Weatherall Institute of Molecular Medicine, University of Oxford, Oxford, UK

* These authors contributed equally to this work.
[‡] These authors jointly supervised work
[#] Corresponding author

SUPPLEMENTARY INFORMATION

SUPPLEMENTARY FIGURES

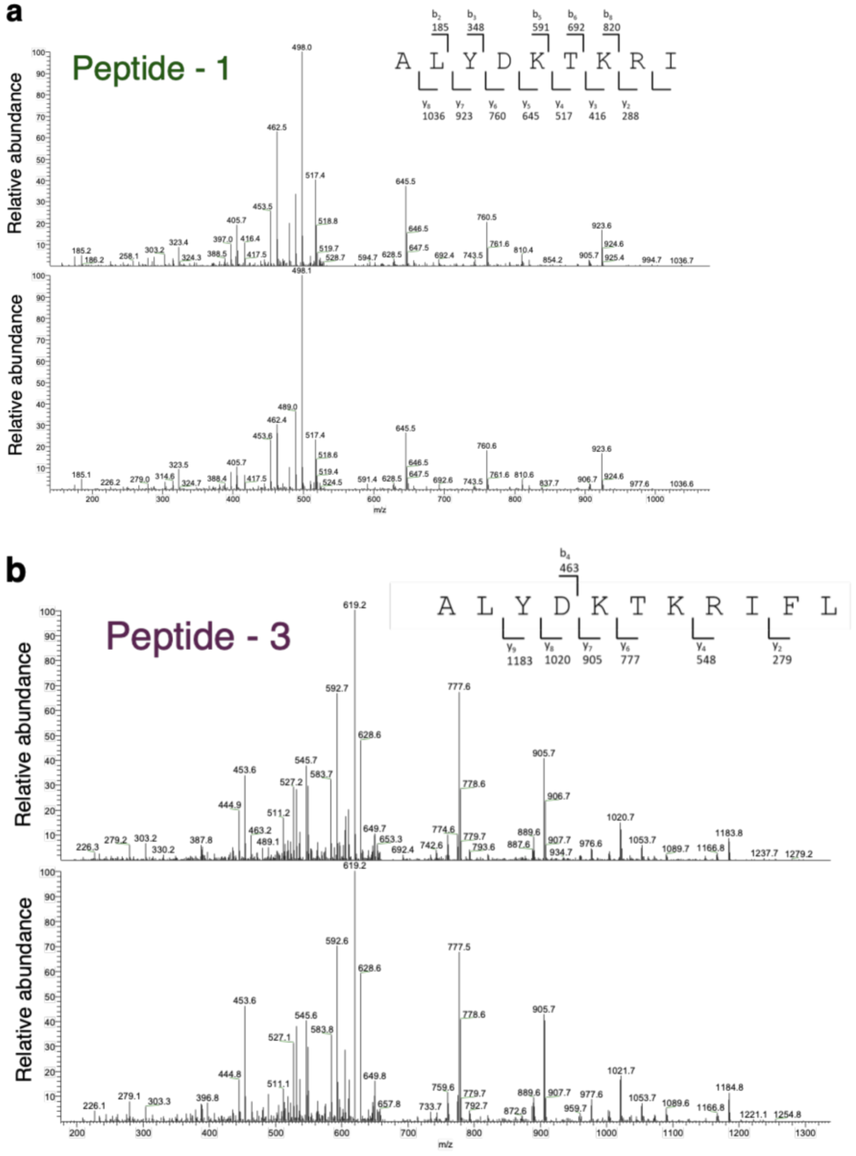

Supplementary Fig. 1: Confirmation of correctly identified peptide-1 and -3

(a,b) Eluted spectra (top) and synthetic peptide spectra (bottom) of peptide-1 (a) and peptide-3 (b) were used to confirm matching fragmentation spectra.

a

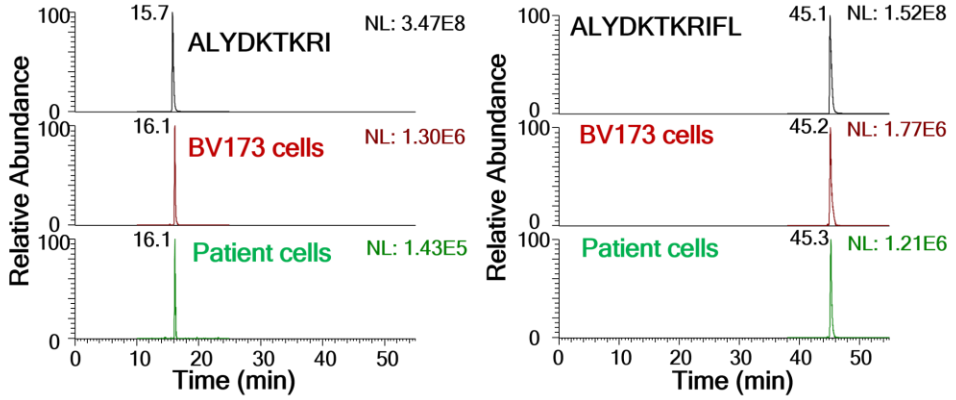

b

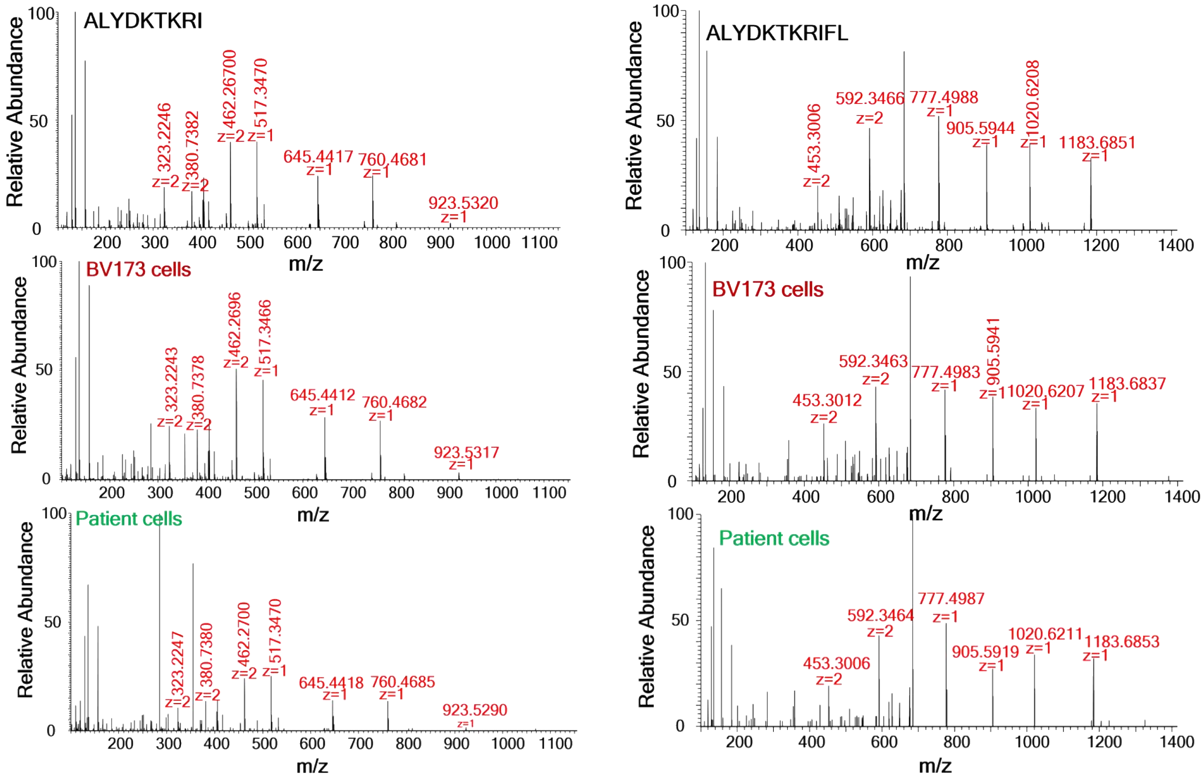

Supplementary Fig. 2: Peptide-1 and peptide-3 can be directly identified from patient leukemia cells

(a,b) Parallel Reaction Monitoring (PRM) analysis, targeting the presence of TdT peptide 1 (m/z=554.3289^{2+}, 369.8889^{3+}) and peptide 3(m/z=684.4044^{2+}, 456.6060^{3+}) in leukemia cells from patient 119N and the leukemia cell line BV173. The extracted ion chromatograms (a) were generated from the ions highlighted in the MS/MS fragmentation spectra (b).

SUPPLEMENTARY TABLES

Supplementary Table 1: HLA-A2-binding peptides derived from TdT

Peptide number	Peptide sequence	Mascot ion score	Observed m/z	Predicted Affinity (nM)*	%Rank *	Predicted binding level*
Pep-1	ALYDKTKRI	45.6	554.3	194.79	1.6	Weak binder
Pep-3	ALYDKTKRIFL	41.4	684.4	115.17	1.2	Weak binder

* Acquired from peptide-MHC class I binding prediction algorithm NetMHC version 4.0

Supplementary Table 2: B-ALL and T-ALL patient characteristics

Pt. ID	Sex	Age at diagnosis	Disease status	Classification	Cytogenetic characteristics	Risk stratification	Tx response	Allo-HSCT*	Outcome
Pt. 2O	M	4	Primary	B-ALL	t(12;21)	Intermediate risk	complete remission	no	alive
Pt.9O	M	5	Primary	B-ALL	t(1;19), hyperdiploidy	Standard risk	complete remission	no	alive
Pt. 20O	F	2	Primary	B-ALL	del(7)(p11), dic(9;20)	Intermediate risk	complete remission	no	alive
Pt. 21O	F	1	Primary	B-ALL	t(12;21)	Intermediate risk	complete remission	no	alive
Pt. 33O	F	7	Primary	B-ALL	Hyperdiploidy	Intermediate risk	complete remission	no	alive
Pt. 40O	F	3	Primary	B-ALL	Normal karyotype	Intermediate risk	complete remission	no	alive
Pt. 1N	F	18	Primary	B-ALL	BCR-ABL1 (minor); 46,XX, der(9)t(1;9)(q12;q12), ider(9)(q10)t(9;22)(q34;q22), der(22)t(q34;q12)	High risk	Complete remission	Yes	Alive
Pt. 9N	F	60	Primary	B-ALL	BCR-ABL1 (minor); 46,XX, t(9;22)(q34;q11)	High risk	Complete remission	No	Alive
Pt. 119N	F	61	Primary**	B-ALL	BCR-ABL	High risk	Refractory	Yes***	Dead
Pt. 32N	M	33	Primary	T-ALL	SIL-TAL1 fusion; 47,XY, t(11;14)(p15;q11)	High risk	Refractory	No	Dead
Pt. 40N	M	17	Primary	T lymphoblastic lymphoma	46,XY	High risk	Complete remission	No	Alive
Pt. 82N	M	28	Primary	T-ALL	46,XY, del(16p21)	High risk	Complete remission	No	Alive
Pt. 4L	M	43	primary	T ALL	t(9;14), t(11;14)	-	-	-	-
Pt. 8L	M	40	primary	T ALL	Normal karytoype	-	-	-	-
Pt. 9L	M	35	primary	T ALL	-	-	-	-	-
Pt. 10L	M	24	Primary	T ALL	Normal karyotype	Standard risk	-	-	-

* Allogeneic hematopeoitic stem cell transplant

**CML in TdT+ lymphoid blasts crisis

***AlloHSCT and stem cell transplant with reduced-intensity conditioning

Supplementary Table 3: TdT and CD19 status of leukemic cells from pediatric B-ALL patients before and after relapse following CD19 CAR T cell therapy

r/r B-ALL

			TdT**		CD19**	
ID	age/gender	Disease status	prior CART	post-CART	prior CART	post-CART
1	8y/f	3rd relapse*	+	+	+	+
2	19y/m	2nd relapse	+	+	+	+
3	15y/m	refractory disease	+	+	+	-
4	9y/m	2nd relapse*	+	+	+	-
5	12y/m	2nd relapse	+	+	+	-

*after allogeneic hematopoietic stem cell transplantation

**leukemic B-ALL blasts, immunophenotyping by flow cytometry

Supplementary Table 4: TdT status of leukemic cells from pediatric T-ALL patient at diagnosis and during disease course

T-ALL

	TdT expression (%)*				
age/gender	Primary diagnosis	Day 15, MRD+	Day 29, MRD+	Relapse	Outcome
13 y/m	100	100	100	100	HSCT, died

*% of leukemic T-ALL blasts, immunophenotyping by flow cytometry

Supplementary Table 5: List of antibodies used in the study with characteristics

CD number/protein target/dye/kit	Fluorochrome	Clone	Species	Supplier	Catalogue number	Dilution factor	Validated research application
CD14	PE-Cy5	61D3	Mouse, anti-human	eBiosciences	15-0149-42	1:160-200	Flow cytometry
HLA-A2	BV650, PE, PE-Cy7	BB7.2	Mouse, anti-human	BioLegend, Diatec	343324, 343306, 343314, 8750	1:200, 1:30	Flow cytometry
CD62L	Pe-Cy7	DREG-56	Mouse, anti-human	BioLegend	304822	1:200	Flow cytometry
CD56	Pe-Cy7	B159	Mouse, anti-human	BD	555515	1:40	Flow cytometry
CD57	BV605	HNK-1	Mouse, anti-human	BioLegend	393303	1:200	Flow cytometry
CD45RO	APC	UCHL1	Mouse, anti-human	BioLegend	304210	1:200	Flow cytometry
CD45RA	PE	HI100	Mouse, anti-human	BD	555489	1:20	Flow cytometry
CCR7	FITC	G043H7	Mouse, anti-human	BioLegend	353216	1:100	Flow cytometry
CD137	PE	4B4-1	Mouse, anti-human	BD, Thermo Fisher Scientific	555956, A51019	1:20, 1:200	Flow cytometry
CD45	AF700, BV605	HI30	Mouse, anti-human	BioLegend	304024, 304042	1:200-600, 1:200	Flow cytometry
TDT	APC	E17-1519	Mouse, anti-human	BD	332791	1:20	Flow cytometry
CD10	PE-Cy7, PE-Cy5, PE	HI10a	Mouse, anti-human	BioLegend	982210, 312206, 312204	1:100-200, 1:300-500, 1:200	Flow cytometry
CD19	BV421,BV785,PE-Cy5	SJ25c1, HI10a, HIB19	Mouse, anti-human	BioLegend	363018, 302239, 302240, 302210	1:200, 1:400	Flow cytometry
CD38	PE/Dazzle594	HIT2	Mouse, anti-human	BioLegend	303538	1:100	Flow cytometry
CD34	APC, BUV395, BV785, PerCP-Cy5.5	8G12, 561, 8G12	Mouse, anti-human	BD, BioLegend, BD	345804, 745727, 343626, 347222	1:200, 1:80, 1:200, 1:20	Flow cytometry
CD1a	PE/Cy7	HI149	Mouse, anti-human	BioLegend	300122	1:50	Flow cytometry
CD2	PE-Cy5	RPA-2.10	Mouse, anti-human	BioLegend	300210	1:300	Flow cytometry
CD3	BB515, PE-Cy5, PerCP-Cy5.5, BV605, FITC, APC-Cy7, BV421, PE-Cy7	HIT3a, SK7, UCHT1	Mouse, anti-human	BD, BioLegend, BD	565100, 30031, 344808, 344836, 344804, 300426, 562426, 300420	1:100-200, 1:300, 1:200, 1.200, 1:200, 1:200, 1:100, 1:200	Flow cytometry
CD8a	APC-Cy7, PE-Cy5, BV421, AF700	RPA-T8, HIT8a	Mouse, anti-human	BioLegend	301016, 301010, 301036, 300920	1:80-300, 1:300, 1:200, 1:200	Flow cytometry
CD4	BUV395, PE-Cy5, BV421	RPA-T4, OKT4	Mouse, anti-human	BD, BioLegend	564724, 300510, 317440	1:300, 1:160-300, 1:200	Flow cytometry
CD5	PE-Cy7, PerCP-Cy5.5	UCHT2, L17F12	Mouse, anti-human	BioLegend	300622, 364006	1:150, 1:200	Flow cytometry
CD7	PE-Cy7, PE	M-T701	Mouse, anti-human	BD	555362, 561934	1:40, 1:200	Flow cytometry
CD33	BV785	WM-53	Mouse, anti-human	BioLegend	303428	1:200	Flow cytometry
CD11b	PE-Cy7	ICRF44	Mouse, anti-human	BioLegend	301308	1:160	Flow cytometry
CD20	PE-Cy7, BV785	2H7	Mouse, anti-human	BioLegend	302308, 302356	1:160-200, 1:200	Flow cytometry

CD235a,b	PE-Cy7	HIR2	Mouse, anti-human	BioLegend	306606	1:500	Flow cytometry
CD99	FITC	DN16	Mouse, anti-human	BIO-RAD	MCA1850F	1:400	Flow cytometry
HLA class I	PE	W6/32	Mouse, anti-human	BioLegend	311406	1:100	Flow cytometry
CD45	BV510, BV605	30-F11	Rat, anti-mouse	BioLegend	103138, 103155	1:400-800, 1:200	Flow cytometry
CD45.1	PE	A20	Mouse, anti-mouse	BioLegend	110708	1:600	Flow cytometry
TCRb	PE	H57-597	Hamster, anti-mouse	BioLegend	109208	1:200	Flow cytometry
Ter119	PE-Cy5	TER-119	Rat, anti-mouse	BioLegend	116210	1:600	Flow cytometry
7AAD						1:200	Flow cytometry
DAPI						1:50000	Flow cytometry
LIVE/DEAD™ Fixable Near-IR				Thermo Fisher Scientific	L10119	1:1000	Flow cytometry
LIVE/DEAD™ Fixable Aqua Dead Cell Stain Kit				Thermo Fisher Scientific	L34957	1:200	Flow cytometry
Cell Trace Violet				Thermo Fisher Scientific	C34557	1:3300	Flow cytometry
5-(and-6)-Carboxyfluorescein Diacetate, Succinimidyl Ester				Thermo Fisher Scientific	C1157	1:2000	Flow cytometry
Alexa Fluor™ 488 NHS Ester	AF488			Invitrogen	A20000		Flow cytometry
Streptavidin	PE, APC			Thermo Fisher Scientific	S866, S868		Flow cytometry
Purified Mouse Anti-Human IFN-γ		NIB42	Mouse, anti-human	BD	551221	1:500	ELISA
Biotin Mouse Anti-Human IFN-γ		4S.B3	Mouse, anti-human	BD	554550	1:500	ELISA
CBA Human Th1/Th2/Th17 Cytokine Kit				BD	560484		ELISA

SUPPLEMENTARY METHODS

Immunopurification of EBV-LCL for identification of TdT-derived peptides presented on HLA

EBV-LCL cells stably transduced with full-length TdT were lysed in 15 ml lysis buffer (50 mM Tris-Cl pH 8.0, 150 mM NaCl, 5 mM EDTA, 0.5% Zwittergent 3-12 (N-dodecyl-N,N-dimethyl-3-ammonio-1-propanesulfonate) and protease inhibitor (Complete, Roche Applied Science)) for 2 h at 0 °C (*1*). Lysates were successively centrifuged to remove nuclei (10 min, 2500 × *g*) and other insoluble material (45 min, 31,000 x *g*). Lysates were passed through a 1.5 ml CL-4B Sepharose column to preclear the lysate and subsequently passed through a 1.5 ml column containing 3.75 mg pan class I (W6/32) IgG coupled to protein A Sepharose (*1*). The W6/32 column was subsequently washed with 5 ml of lysis buffer, 5 ml of low salt buffer (20 mM Tris-Cl pH 8.0, 120 mM NaCl), 1 ml of high salt buffer (20 mM Tris-Cl pH 8.0, 1 M NaCl), and finally with 5 ml of low salt buffer. HLA α chain, β2m and peptides were eluted with 5 ml of 10% acetic acid, and filtered through 10 kDa filters (PAL incorporation) to remove HLA protein chains. The filtrate was purified by solid phase extraction (SPE) (Oasis HLB, Waters).

The SPE purified material was fractionated on a homemade SCX column (320 µm inner diameter, 15 cm, polysulfoethyl A 3 µm, Poly LC) run at 4 µl/min. Gradients were run for 10 min at 100% solvent A (100% water/0.1% TFA), after which a linear gradient started to reach 100% solvent B (250 mm KCl, 35% ACN/0.1% TFA) over 15 min, followed by 100% solvent C (500 mm KCl, 35% ACN/0.1% TFA) over the next 15 min. The gradient remained at 100% solvent C for 5 min and then switched again to 100% solvent A. Twenty 4-µl fractions were collected in vials pre-filled with 100 µl 95/3/0.1 water/ACN/FA v/v/v.

LC-MS/MS Analysis

The SCX fractions were analyzed via on-line nano-HPLC-MS with a system consisting of a conventional Agilent 1100 gradient HPLC system (Agilent, Waldbronn, Germany), as described by Meiring et al. (*2*) and an LTQ-FT Ultra mass spectrometer (Thermo, Bremen, Germany). Fractions were injected onto a homemade precolumn (100 µm × 15 mm; Reprosil-Pur C18-AQ 3

µm, Dr. Maisch, Ammerbuch, Germany) and eluted via a homemade analytical nano-HPLC column (15 cm × 50 µm; Reprosil-Pur C18-AQ 3 um). The gradient was run from 0% to 50% solvent B (10/90/0.1 water/ACN/FA v/v/v) over 20 min. The nano-HPLC column was drawn to a tip of ∼5 µm and acted as the electrospray needle of the MS source. The mass spectrometer was operated in data dependent mode, automatically switching between MS and MS/MS acquisition. Full scan mass spectra were acquired in a Fourier transform ion cyclotron resonance mass spectrometer with a resolution of 25,000 at a target value of 5,000,000. The two most intense ions were then isolated for accurate mass measurements by a selected ion monitoring scan in Fourier transform ion cyclotron resonance with a resolution of 50,000 at a target accumulation value of 50,000. The selected ions were then fragmented in the linear ion trap using collision-induced dissociation at a target value of 10,000.

The tandem mass spectra were matched against the homo sapiens canonical (70,000 entries) database using the Mascot search engine (version 2.2.04, Matrix Science, London, UK) with a precursor mass tolerance of 2 ppm, with methione oxidation and cysteinyl on cysteine as variable modifications and a product ion tolerance of 0.5 Da.

In a post-analysis process, raw data were first converted to peak lists using Proteome Discoverer version 2.2 (Thermo Electron), and then submitted to the Uniprot Homo sapiens database (71,591 entries), using Mascot v. 2.2.07 (www.matrixscience.com) for protein identification. Mascot searches were with 2 ppm and 0.5 Da deviation for precursor and fragment mass, respectively, and no enzyme was specified. Methionine oxidation and cysteinyl on cysteine were set as variable modifications. Peptides with MASCOT scores <35 were generally discarded. Tandem mass spectra of TdT derived peptides were manually inspected. The correct identification of the TdT derived peptides was confirmed by comparison of the eluted and synthetic tandem mass spectra.

Immunoprecipitation-Targeted Mass spectrometry (IP-MS) analysis of TdT peptides presented on HLA of leukemia cells

The leukemia cell line BV173 cells (350 Million) or leukemia cells from patient 119N (450 Million) were lysed in PBS containing 1% lauryl maltoside, 1mM EDTA, 1mM PMSF and Sigma protease inhibitors (1:200) for 1 hour at 4°C. The clarified cell lysates (12.000 RPM, 30 minutes) were added to 500uL of AminoLink plus bead slurry (ThermoFisher Scientific) coated with pan HLA class I specific antibody (W6/32, BioXcell) and incubated for 3 hours at 4°C. Following the manufacturers suggestions, 10mg of W6/32 antibody was coupled to 2 ml of AminoLink plus bead slurry. After a 3-hour incubation with cell lysates, the beads were washed with 3 mL of 0.1M Tris-HCl/150mM NaCl, 3 ml of 0.1M Tris-HCl/400mM NaCl, and again with 3 mL of 0.1M Tris-HCl/150mM NaCl and finally with 3 mL of 0.1 M Tris-HCl. The HLA bound peptides were then eluted with 1 mL of 1% TFA, followed by another two elutions, each with 1mL of 1% TFA. The peptide elutions were pooled and then loaded on to 10kDa molecular weight cutoff filters for removal of B2M and heavy chain. The filtrate was then loaded on to the Discovery DSC-C18 SPE column for desalting of the peptides before MS analysis. The peptides were vacuum concentrated and dissolved in 25 uL of 3% acetonitrile containing 0.1% TFA. The peptide solution (5 uL) was analyzed using an Ultimate 3000 nano-UHPLC system (Dionex, Sunnyvale, CA, USA) connected to a Q Exactive mass spectrometer (ThermoElectron, Bremen, Germany) equipped with a nano electrospray ion source. For liquid chromatography separation, an Acclaim PepMap 100 column (C18, 3 µm beads, 100 Å, 75 µm inner diameter) capillary of 50 cm bed length was used. A flow rate of 300 nL/min was employed with a solvent gradient of 10-35% B in 95 min, to 50% B in 13 min and then to 80% B in 2 min. Solvent A was 0.1% formic acid and solvent B was 0.1% formic acid/90% acetonitrile. The mass spectrometer was operated in Parallel Reaction Monitoring (PRM) mode, to specifically target the presence of TdT peptide 1 (m/z= 554.3289^{2+}, 369.8889^{3+}), eluting within a retention time window of 10-25 minutes, as determined using synthetic analog. TdT peptide 3 (m/z= 684.4044^{2+}, 456.6060^{3+}) was targeted within a retention time window of 38-55 minutes. The MS/MS spectra using higher-energy collision induced dissociation (HCD) were acquired with the resolution R = 35,000, after accumulation to a target of 2e5. The normalized collision energy was set to NCE 27, isolation window was m/z = 1.7 with an offset of 0.3m/z. The maximum allowed ion accumulation for the MS/MS spectrum was 120ms. Raw data were analyzed using the Xcalibur™ software.

REFERENCES

1. C. Hassan *et al.*, The human leukocyte antigen-presented ligandome of B lymphocytes. *Mol Cell Proteomics* **12**, 1829-1843 (2013).
2. H. D. Meiring, E. van der Heeft, G. J. ten Hove, A. P. J. M. de Jong, Nanoscale LC–MS(n): technical design and applications to peptide and protein analysis. *Journal of Separation Science* **25**, 557-568 (2002).
