# Supplementary material for: T cells targeted to TdT kill leukemic lymphoblasts while sparing normal lymphocytes
Source: Nat Biotechnol. 2021 Dec 6;40(4):488–98. doi: 10.1038/s41587-021-01089-x (PMC9005346; doi:10.1038/s41587-021-01089-x)
Supplement: Supplementary file 1 [file 41587_2021_1089_MOESM2_ESM.pdf]

nature research

Corresponding author(s): Johanna Olweus

Last updated by author(s): 17/08/21

Reporting Summary

Nature Research wishes to improve the reproducibility of the work that we publish. This form provides structure for consistency and transparency in reporting. For further information on Nature Research policies, see our Editorial Policies and the Editorial Policy Checklist.

Statistics

For all statistical analyses, confirm that the following items are present in the figure legend, table legend, main text, or Methods section.

n/a	Confirmed	
☐	☒	The exact sample size (*n*) for each experimental group/condition, given as a discrete number and unit of measurement
☐	☒	A statement on whether measurements were taken from distinct samples or whether the same sample was measured repeatedly
☐	☒	The statistical test(s) used AND whether they are one- or two-sided<br>*Only common tests should be described solely by name; describe more complex techniques in the Methods section.*
☒	☐	A description of all covariates tested
☐	☒	A description of any assumptions or corrections, such as tests of normality and adjustment for multiple comparisons
☐	☒	A full description of the statistical parameters including central tendency (e.g. means) or other basic estimates (e.g. regression coefficient) AND variation (e.g. standard deviation) or associated estimates of uncertainty (e.g. confidence intervals)
☐	☒	For null hypothesis testing, the test statistic (e.g. *F*, *t*, *r*) with confidence intervals, effect sizes, degrees of freedom and *P* value noted<br>*Give P values as exact values whenever suitable.*
☒	☐	For Bayesian analysis, information on the choice of priors and Markov chain Monte Carlo settings
☒	☐	For hierarchical and complex designs, identification of the appropriate level for tests and full reporting of outcomes
☐	☒	Estimates of effect sizes (e.g. Cohen's *d*, Pearson's *r*), indicating how they were calculated

Our web collection on statistics for biologists contains articles on many of the points above.

Software and code

Policy information about availability of computer code

Data collection	1. Flow cytometry was performed on BD LSR II Cytometer (BD Biosciences) and data was acquired with the help of BD FACSDIVA V8.0.1 software.<br>2. Cell sorting was performed with SH800 (Sony Biotechnology), BD FACSAria II (BD Biosciences) or BD FACS AriaFusion (BD Biosciences) cell sorter.<br>3a. High performance liquid chromatography (HPLC) and mass spectrometry (MS) data was acquired by a system consisting of Agilent 1100 gradient HPLC system (Agilent) and an LTQ-FT Ultra mass spectrometer (Thermo Scientific).<br>3b. Immunoprecipitation- targeted Mass spectrometry (IP-MS) data was acquired using an Ultimate 3000 nano-UHPLC system (Dionex, Sunnyvale, CA, USA) connected to a Q Exactive mass spectrometer (ThermoElectron, Bremen, Germany) equipped with a nano electrospray ion source.<br>4. Bioluminescence imaging of mice was performed with IVIS Spectrum in vivo imaging system (PerkinElmer).
Data analysis	1. Flow cytometry data were analyzed with FlowJo versions 9 and 10.<br>2. Numerical data were statistically analyzed and graphs were generated with the help of GraphPad Prism versions 6, 7 and 8 software.<br>3a. The tandem mass spectra were matched against the homo sapiens canonical (70,000 entries) database using the Mascot search engine (version 2.2.04, Matrix Science, London, UK, www.matrixscience.com). Post-analysis of the HPLC-MS data was performed with Proteome Discoverer version 2.2 (Thermo Electron), and then submitted to the Uniprot Homo sapiens database (71591 entries), using Mascot v. 2.2.07 (www.matrixscience.com) as described in the methods section.<br>3b. Raw data from targeted MS analysis were analyzed using the XcaliburTM software version 4.1.<br>4. Bioluminescence imaging was analyzed with Living image software version 4.5.2 (PerkinElmer)<br>5. Peptide-MHC complex models were visualized by the PyMOL molecular visualization system (version 2.3). Peptide (ILAKFLHTL) from human telomerase reverse transcriptase (PDB accession code: 5MEQ) and (FVLELEPEWTV) derived from Toxoplasma gondii (PDB accession code: 5D9S) were utilized to generate peptide-1 and -3 HLA-A2 models respectively as described in the methods section.<br>6. Curated human proteome databases UniProtKB/Swiss-Prot and Protein Data Bank were queried by employing ScanProsite tool (https://

prosite.expasy.org/scanprosite/).

For manuscripts utilizing custom algorithms or software that are central to the research but not yet described in published literature, software must be made available to editors and reviewers. We strongly encourage code deposition in a community repository (e.g. GitHub). See the Nature Research guidelines for submitting code & software for further information.

Data

Policy information about availability of data

All manuscripts must include a data availability statement. This statement should provide the following information, where applicable:

- Accession codes, unique identifiers, or web links for publicly available datasets
- A list of figures that have associated raw data
- A description of any restrictions on data availability

The data that support the findings of this study are included in the manuscript and in supplementary information. Source data are also provided. Additionally, datasets used in the study are: Protein Data Bank (accession codes: 5MEQ, https://www.rcsb.org/structure/5MEQ and 5D9S, https://www.rcsb.org/structure/5D9S), Homo sapiens canonical database using the Mascot search engine (version 2.2.04, www.matrixscience.com), Uniprot Homo sapiens database, using Mascot v. 2.2.07, www.matrixscience.com), curated human proteome databases UniProtKB/Swiss-Prot and Protein Data Bank by ScanProsite tool (https://prosite.expasy.org/scanprosite/), peptide-MHC class I binding prediction algorithm NetMHC version 4.0 (http://www.cbs.dtu.dk/services/NetMHC/)

Field-specific reporting

Please select the one below that is the best fit for your research. If you are not sure, read the appropriate sections before making your selection.

☒ Life sciences ☐ Behavioural & social sciences ☐ Ecological, evolutionary & environmental sciences

For a reference copy of the document with all sections, see nature.com/documents/nr-reporting-summary-flat.pdf

Life sciences study design

All studies must disclose on these points even when the disclosure is negative.

Sample size

In experiments with xenograft B-ALL cell line models, we expected to see 25% decrease in the tumor bioluminescence signal in the treated animals compared to the control T-cell treated mice. In order to reach a p value of 0.05 with 95% power, we chose to have at least 7 mice per group. We used this as a guideline to choose numbers of mice per T cell treatment group (DMF5 or T3) in primary human B-ALL xenograft model (to observe therapeutic efficacy) and humanized mouse model engrafted with HLA-A2pos human cord blood cells (to observe lack of toxicity) without employing power calculations. Samples sized were expected to be sufficient based upon our previous observations with cell line based B-ALL models.

Data exclusions

In experiments using the primary human B-ALL xenograft model, one mouse that already showed signs of poor health and weight loss prior to T-cell injections, had to be euthanized 2 days post treatment according to the guidelines from institutional animal experimentation ethics committee and excluded prior to any post-treatment analysis. For the experiments using the humanized mouse model engrafted with HLA-A2pos human cord blood cells, one mouse was excluded due to the reconstitution failure of B cell lineage prior to T-cell treatment, according to the pre-determined exclusion criteria.

Replication

Replication of experiments is described in detail in all figure legends. Briefly, survival and tumor bioluminescence imaging data from two independent experiments were pooled and shown in the manuscript. Two independent experiments were performed using the primary human B-ALL xenograft model with very similar results. One experiment was performed using the humanized mouse model engrafted with HLA-A2pos human cord blood cells.
ALL patient samples were tested at least twice to verify reproducibility and all technical replicates are shown for one representative experiment. The experiment shown in Fig. 2g was performed once, but the functional analysis was performed with two independent methods with different readouts (i) ELISA (IFN-gamma secretion) and (ii) flow cytometric measurements of T cell activation by CD137 upregulation. Results from both T-cell functional readouts correlated strongly with each other, as shown in the manuscript in Extended Data Fig. 2c.

Randomization

In experiments with xenograft B-ALL cell line models after tumor injection, mice were randomly assigned in different groups one day before T cell therapy. There was no difference in the tumor BLI signal between experimental groups before start of therapy (by one-way ANOVA with Tukey's multiple comparison test).
There is generally a relatively large variation in the tumor/human cell engraftment in the primary human B-ALL xenograft model and in the humanized mouse model engrafted with HLA-A2pos human cord blood cells. Therefore mice were assigned to different groups based upon their pre-treatment tumor burden or human cell engraftment in the bone marrow/blood to ensure that experimental groups had similar means prior to T-cell treatment. For the in vitro experiments, randomization was not performed as it is not applicable and is not generally performed in the field.

Blinding

Colonies generated from sorted normal adult human BM CD34+ cells were counted blindly. The investigators were not blinded during group allocation or analysis in mice experiments. Blinding would be impractical due to the limitations related to manpower, and because of the potential alloreactivity that needed to be closely assessed. Due to the nature of the other in vitro experiments, blinding was not possible and is not generally performed in the field as data acquisition is quantitative (flow cytometry or MS) rather than qualitative, and therefore less influenced by observer bias.

Reporting for specific materials, systems and methods

We require information from authors about some types of materials, experimental systems and methods used in many studies. Here, indicate whether each material, system or method listed is relevant to your study. If you are not sure if a list item applies to your research, read the appropriate section before selecting a response.

Materials & experimental systems

n/a	Involved in the study
☐	☒ Antibodies
☐	☒ Eukaryotic cell lines
☒	☐ Palaeontology and archaeology
☐	☒ Animals and other organisms
☐	☒ Human research participants
☒	☐ Clinical data
☐	☒ Dual use research of concern

Methods

n/a	Involved in the study
☒	☐ ChIP-seq
☐	☒ Flow cytometry
☒	☐ MRI-based neuroimaging

Antibodies

Antibodies used

The following fluorescently conjugated anti-human antibodies were acquired from BD Biosciences or BioLegend unless otherwise specified: Anti-HLA-A2 (BB7.2), -CD62L (DREG-56), CD56 (HCD56, B159), -CD57 (HNK-1), -CD45RO (UCHL1), -CD45RA (HI100), -CCR7 (150503), -CD137 (4B4-1), -CD45 (HI30), -TdT (E17-1519), -CD10 (HI10a), -CD19 (HIB19, SJ25C1), -CD38 (HIT2), -CD34 (581, 8G12), -CD1a (HI149), -CD2 (S5.2, RPA-2.10), -CD3 (UCHT1, OKT3, HIT3a), -CD8a (RPA-T8), -CD4 (RPA-T4), -CD5 (UCHT2, L17F12), -CD7 (M-T701), -CD33 (WM-53), -CD11b (ICRF44),-CD14 (61D3), -CD20 (clone 2H7), -CD56 (B159), -CD235a,b (HIR2), -CD99 (DN16, Bio Rad), -CD3 (SK7, eBioscience), -CD14 (HCD14, 61D3 eBioscience), anti-pan HLA class I antibody (W6/32). Anti-mouse antibodies: CD45 (30-F11), TCRβ chain (H57-597) and Ter119 (TER-119). Live/Dead Fixable Near-IR Dead Cell Stain kit (Life Technologies), 7AAD (Sigma Aldrich) or DAPI (Thermofisher) were used to exclude dead cells in all flow cytometry experiments. The following antibodies for ELISA were acquired from BD Pharmingen or R&D Systems: mouse anti-human IFN-γ capture antibody (NIB42) and Biotin Mouse Anti-Human IFN-γ detection antibody (4S.B3). Supplementary Table 5 lists all antibodies with supplier, species, catalogue number, clone name, fluorochromes, validated research application and dilution factor used.

Validation

All antibodies used in the study are available commercially and have been validated by commercial suppliers for use in diagnostics/research on samples generated from humans or mice, and for flow cytometry or ELISA as applicable. Detailed information is provided in Supplementary Table 5.

Eukaryotic cell lines

Policy information about cell lines

Cell line source(s)

The following cell lines were used in the study: NALM-6, BV173, EBV-LCL, HPB-ALL, T2, REH, RD, U-2 OS, FM-6, HeLa, HaCaT, COLO688, EA.hy926, U-87 MG, Daoy, HCT-116, CHP-212, MCF7, K562, RS4;11, Phoenix AMPHO. Cell lines were obtained from American Tissue Culture Collection (ATCC) and Deutsche Sammlung von Mikroorganismen und Zellkulturen (DSMZ). EBV-LCL cell lines were generated in-house by immortalizing human PBMC's from HLA-A2 positive and negative donors with Epstein-Barr viral supernatants. Epstein-Barr viral supernatants were produced in B95-8 cell line.

Authentication

Authenticated cell lines were purchased from ATCC and DSMZ (RS4;11, T2, RD, K562, EA.hy926, Daoy, HCT-116, CHP-212, Phoenix AMPHO), and cryopreserved aliquots labeled according to passage. Only low passages (1-4 passages) were used to start cultures. The identity of the passage used (5 or higher) experimentally of the cell lines NALM-6, BV173, REH, HPB-ALL, U-2 OS, FM-6, HeLa, HaCaT, MCF7, COLO 688, U-87 MG was ascertained by short tandem repeat DNA profiling, a service provided by Labcorp DNA Identification Lab, NC, USA (formerly Genetica, https://celllineauthentication.com/). In-house immortalized EBV-LCL cells were regularly tested for CD20 or CD19 staining to confirm their B cell origin.

Mycoplasma contamination

Cells were tested regularly for mycoplasma contamination, and were confirmed negative before experimental use.

Commonly misidentified lines
(See ICLAC register)

HPB-ALL was used as a T-ALL cell line with desirable characteristics (TdT pos, HLA-A2 neg). Although registered in the ICLAC database as a commonly misidentified cell-line, we acquired an authenticated cell line from DSMZ stock (ACC-483). The ICLAC database also states that DSMZ has a correct version of non-contaminated cell line.

Animals and other organisms

Policy information about studies involving animals; ARRIVE guidelines recommended for reporting animal research

Laboratory animals

For establishing xenograft B-ALL cell line models, 8- to 10-weeks-old female and male NOD-scid IL2Rgnull (NSG) mice were used.
To establish primary human B-ALL xenografted mice, 9-15 weeks old female NOD.Cg-Prkdcscid Il2rgtm1Wjl/SzJ (NSG; Jackson Laboratory stock 005557) was used. Female NSG mice stably engrafted with HLA-A2pos human cord blood cells were purchased from the Jackson laboratory at 27 weeks of age (transplanted at 3 weeks of age).
Mice were housed in IVC-Mouse GM 500 cages with a light cycle of 4 a.m – 4 p.m in 21°C with 50% humidity.

Wild animals

No wild animals were used.

Field-collected samples

No field-collected samples were used.

Ethics oversight	Experiments were performed according to the guidelines and obtained permissions from the ethics committees at Stockholm Norra Djurförsöksetisks Nämd (17978-2018) and Norwegian Food Safety Authority (17500)

Note that full information on the approval of the study protocol must also be provided in the manuscript.

Human research participants

Policy information about studies involving human research participants

Population characteristics	Patient demographics, disease characteristics and sample characteristics have been shown in the manuscript (Supplementary Tables 2, 3 and 4).
Recruitment	Pediatric and young adult relapsed/refractory (r/r) B-ALL patients were enrolled into and treated according to the Chimeric Antigen Receptor (CAR) T cell trials ClinicalTrials.gov Identifier NCT02435849 and NCT03123939; pediatric T-ALL patients according to NOPHO-ALL-2008 (NCT00816049). Institutional Review Board and ethical approvals to use primary human diagnostic blood and bone marrow samples, from pediatric and adult patients were obtained, as were informed written consent from patients or their guardians. Patient samples were selected for inclusion in our study after reviewing available diagnostic information from institutional biobanks. TdT and HLA-A2 status was determined as part of the diagnostic workup, and confirmed by flow cytometric measurements in our experiments. There was no self-selection bias. PB mononuclear cells (PBMCs) from healthy donor buffy coats were obtained from the blood bank of Oslo University Hospital, and PB or bone marrow mononuclear cells from leukemia patients were from biobanked, cryopreserved material (ethical approval numbers: REK 2018/879, REK 2018/1246).<br>Thymocytes were isolated from human thymus removed as a consequence of routine procedure for open cardiac surgery to correct a congenital cardiac defect (in an otherwise healthy child) following informed written consent from guardians and ethical approval (ethical approval number: REK 2019/31516). PB mononuclear cells (PBMCs) from healthy donor buffy coats were obtained from the blood bank of Oslo University Hospital.<br>Bone marrow mononuclear cells were obtained from four HLA-A2pos healthy donors collected at the Karolinska University Hospital with informed consent and ethical approval (EPN 2018/901-31).
Ethics oversight	This study was approved by the Regional Committee for Medical and Health Research Ethics (REC) South-East, Norway (2018/879, 2018/1246, 2019/31516), the Institutional Review Board and the Data Protection Officer, Oslo University Hospital, Swedish Ethical Review Authority, Stockholm (EPN 2018/901-31), and performed in accordance with the Declaration of Helsinki.

Note that full information on the approval of the study protocol must also be provided in the manuscript.

Dual use research of concern

Policy information about dual use research of concern

Hazards

Could the accidental, deliberate or reckless misuse of agents or technologies generated in the work, or the application of information presented in the manuscript, pose a threat to:

No	Yes	
☒	☐	Public health
☒	☐	National security
☒	☐	Crops and/or livestock
☒	☐	Ecosystems
☒	☐	Any other significant area

Experiments of concern

Does the work involve any of these experiments of concern:

No	Yes	
☒	☐	Demonstrate how to render a vaccine ineffective
☒	☐	Confer resistance to therapeutically useful antibiotics or antiviral agents
☒	☐	Enhance the virulence of a pathogen or render a nonpathogen virulent
☒	☐	Increase transmissibility of a pathogen
☒	☐	Alter the host range of a pathogen
☒	☐	Enable evasion of diagnostic/detection modalities
☒	☐	Enable the weaponization of a biological agent or toxin
☒	☐	Any other potentially harmful combination of experiments and agents

Flow Cytometry

Plots

Confirm that:

- [x] The axis labels state the marker and fluorochrome used (e.g. CD4-FITC).
- [ ] The axis scales are clearly visible. Include numbers along axes only for bottom left plot of group (a 'group' is an analysis of identical markers).
- [ ] All plots are contour plots with outliers or pseudocolor plots.
- [x] A numerical value for number of cells or percentage (with statistics) is provided.

Methodology

Sample preparation	Samples analyzed by flow cytometry contained mononuclear cells in suspension isolated from buffy coats by density gradient centrifugation that were cultured with standard media as detailed in the manuscript. Patient blood or bone marrow samples were harvested for diagnostic purposes and mononuclear cells were isolated by leukapheresis. Samples were then cryopreserved and stored in liquid nitrogen in designated cell biobanks in our institutions before use in the experiments as detailed in the methods. Cell lines utilized were cultured for variable amounts of time in recommended media prior to experiments.<br>Blood samples and bone marrow were harvested from murine xenograft B-ALL cell line models. RBC lysis was performed by ACK lysis buffer, followed by washing and surface or intracellular staining with different antibodies for flow cytometry analysis.<br>Peripheral blood, spleen, thymus and bone marrow was harvested from the primary human B-ALL xenograft model or humanized mouse model engrafted with HLA-A2pos human cord blood cells and prepared into single cell suspensions in PBS supplemented with fetal calf serum. All samples were incubated with Fc-Block prior to staining with monoclonal antibodies.
Instrument	BD LSR II (BD Biosciences) equipped with high throughput sampler (HTS). Cell sorting was performed with SH800 (Sony Biotechnology), BD FACSAria II or BD FACS AriaFusion (BD Biosciences) cell sorter.
Software	For data collection for all experiments, BD FACSDiva V8.0.1 was used. For data analysis FlowJo versions 9 and 10 were used.
Cell population abundance	Single cytotoxic T cells were sorted by FACS and expanded into T cell clones. Purity was confirmed with relevant peptide-MHC tetramer staining after (86% - 100% purity). Tumor cell lines that were transduced with HLA-A2 or GFP and firefly luciferase, were purified by FACS (purity > 95%). CD34+ cells used for CFC culture were sorted on a BD FACS AriaFusion (purity > 95%).

Gating strategy

For all flow cytometry experiments FSC-A/SSC-A was used for gating of mononuclear cells. Doublets were excluded. 7AAD, DAPI or Live/dead fixable near-IR -positive cells were gated out to exclude non-viable cells.

For pMHC multimer staining: From the live cell gate, CD8+ events were gated and subsequently, pMHC multimer+ events were identified as double positive for PE and APC conjugated pMHC multimers.

For flow cytometry-based cytotoxicity assays and T cell activation assays, tumor cell lines and transduced T cells were stained with surface antibodies and were gated as detailed in the methods and shown in Extended Data figures 4 and 8. Primary patient samples were also stained with surface antibodies for the presence of tumor blasts, mature T/B cells or CD34+lin- cells. Effector cells in the same well were pre-labeled with CellTrace Violet (CTV, Life Technologies) for separation from live target cells. Tumor blasts, mature B and T cells, and CD34+lin- cells were gated and overlaid to be visualized as T-Distributed Stochastic Neighbor Embedding (tSNE) plots. CountBright Absolute Counting Beads were utilized to acquire equal numbers of events in each tested well.

To analyze presence of transduced T cells in blood and bone marrow of xenograft B-ALL cell line models, single live cells were gated as described in methods. Total leukocytes were defined as antigen positive for both human and mouse CD45. From CD45+ gate, human TCR-transduced T cells were identified as anti-human CD3+, CD8+ and anti-mouse TCR-β+. The murine constant part introduced into the TCR-β chain served as a reporter for TCR transduction.

For the flow cytometric analysis of thymocytes, samples from normal human thymus removed from a four months old child with congenital cardiac defect were used. Flow plots displaying the gating strategy to identify the four key developmental stages during thymocyte differentiation (early double negative, late double negative, double positive and single positive) are shown in Extended data Fig. 9.

To generate the patient-derived xenograft model, viable T cell-depleted bone marrow cells from an HLA-A2pos B-ALL patient was identified through 7AAD and CD3 exclusion and yield sorted for transplantation. Upon termination, peripheral blood, bone marrow and spleen was subjected to detailed flow cytometry analysis using anti-human CD45, -CD8a, -CD4, -CD3, -CD19, -HLA-A2, -CD10 and anti-mouse CD45, -Ter119 and -TCRβ. The same panel was used to monitor the engraftment levels before and after T cell infusion.

To investigate the impact of T3 cells on normal human hematopoiesis in humanized NSG mice the persistence of infused T cells was monitored in PB, and impact of therapy on mature blood cell lineages was monitored in PB, spleen, thymus and bone marrow by flow cytometry with anti-human CD45, -CD8a, -CD19, -CD33, -CD4, -CD3 and anti-mouse CD45, -Ter119 and TCRβ. The impact on human T cell progenitors in mouse thymus was investigated through surface and intracellular staining with anti-human TdT, -CD45, -CD8 -CD19, -CD3 (intracellular and surface), -CD4, -HLA-A2 and anti-mouse CD45.

☒ Tick this box to confirm that a figure exemplifying the gating strategy is provided in the Supplementary Information.
